# Supplementary material for: A general supramolecular strategy for fabricating full-color-tunable thermally activated delayed fluorescence materials
Source: Nat Commun. 2024 Feb 16;15:1425. doi: 10.1038/s41467-024-45717-x (PMC10873404; doi:10.1038/s41467-024-45717-x)
Supplement: Supplementary file 1 — Supplementary Information [file 41467_2024_45717_MOESM1_ESM.pdf]

Supplementary information

A general supramolecular strategy for fabricating full-color-tunable thermally activated delayed fluorescence materials

Nan Xue[1,3], He-Ye Zhou[2,3], Ying Han[2], Meng Li[1,2], Hai-Yan Lu[1,*] & Chuan-Feng Chen[1,2,*]

[1]University of Chinese Academy of Science, Beijing 100049, China

[2]Beijing National Laboratory for Molecular Science, CAS Key Laboratory of Molecular Recognition and Function, Institute of Chemistry, Chinese Academy of Science, Beijing 100190, China

[3]These authors contributed equally: Nan Xue, He-Ye Zhou.

*Corresponding authors emails: haiyanlu@ucas.ac.cn; cchen@iccas.ac.cn

Contents

1. Supplementary Methods 3

1.1 Theoretical Calculation Methods 3

1.2 Synthesis of Calix[3]acridan 3

1.3 Preparation of Cocrystals 6

2. Supplementary Discussion 9

2.1 Frontier Orbital Calculations of G1~G7...9

2.2 Crystal Structures and Crystal Data 10

2.3 Powder X-ray Diffraction Analysis 25

2.4 Thermogravimetic Analysis and Differential Scanning Calorimetry 26

2.5 Physical Properties 27

2.6 Hole-electron Analysis of G1@C[3]A~G7@C[3]A 38

3. Supplementary References 39

1. Supplementary Methods

1.1 Theoretical calculation methods

The theoretical calculations of C[3]A, guests (G1~G7), and cocrystals were executed by the Gaussian 09 software package.[1] The optimized ground state geometries of C[3]A and guests (G1~G7) were calculated with the B3LYP functional[2] and 6-31g(d) basis set.[3] The ESP maps were visualized by the GaussView 5.0 program after optimized ground state geometries of C[3]A and guests (G1~G7). The ground state geometry optimizations of cocrystals based on density functional theory[4] (DFT) were performed with PBE0[5] functional and the 6-31g(d) basis set. The excited state calculations of cocrystals based on time-dependent density functional theory[6] (TD-DFT) were performed with PBE0 functional and the 6-311g(d) basis set.[7] The frontier molecular orbitals of C[3]A, guests (G1~G7), and cocrystals were generated by the Multiwfn 3.8 program[8] based on the optimized ground state geometries and visualized with the VMD program[9]. The IGM analysis[10,11] was carried out on the Multiwfn 3.8 program based on the optimized ground state geometries through subfunction 10 of main function 20 (visual study of weak interactions) and visualized by the VMD program. Hole-electron analysis[12] was calculated based on TD-DFT calculation, the Multiwfn 3.8 program through main function 18 (electron excitation analysis), and visualized by the VMD program.

1.2 Synthesis of calix[3]acridan

The 9,9-mimethyl-9,10-dihydroacridine (4.0 g, 19.1 mmol) and sodium hydroxide (1.6 g, 40 mmol) were mixed in DMSO (45 mL), then iodomethane (1.8 mL, 28.9 mmol) was added and the mixture was stirred at room temperature for 24h. The reaction was subsequently quenched with water (150 mL) and the organic phase was extracted with dichloromethane. The organic phase was purified by column chromatography (CH_2Cl_2/petroleum ether, 1:8 v/v) to afford 9,9,10-trimethyl-9,10-dihydroacridine as white solid (4.0 g, 94%). Subsequently, $FeCl_3{\cdot}6H_2O$ (242 mg, 0.9 mmol) was added to a

solution of 9,9,10-trimethyl-9,10-dihydroacridine (2.00 g, 8.96 mmol) and paraformaldehyde (807 mg, 26.9 mmol) in anhydrous dichloromethane (900 ml), and stirred at room temperature for 18 hours. The organic phase was purified by column chromatography (CH_2Cl_2/petroleum ether, 1:2 v/v) to afford calix[3]acridan (1.9 g, 90%) as a white solid.[13] ^{1}H NMR (400 MHz, $CDCl_3$): δ 7.12 (d, J = 10.1 Hz, 6H), 6.98 (s, 6H), 6.81 (d, J = 8.0 Hz, 6H), 3.91 (s, 6H), 3.39 (s, 9H), 1.27 (s, 18H). ^{13}C NMR (101 MHz, $CDCl_3$): δ 140.7, 133.6, 133.0, 126.7, 123.6, 111.6, 77.0, 41.1, 36.6, 33.3, 26.9. HRMS (ESI): *m/z* $[M + H]^+$ = 706.4153 (calcd. 706.4156 for $C_{51}H_{52}N_3^+$).

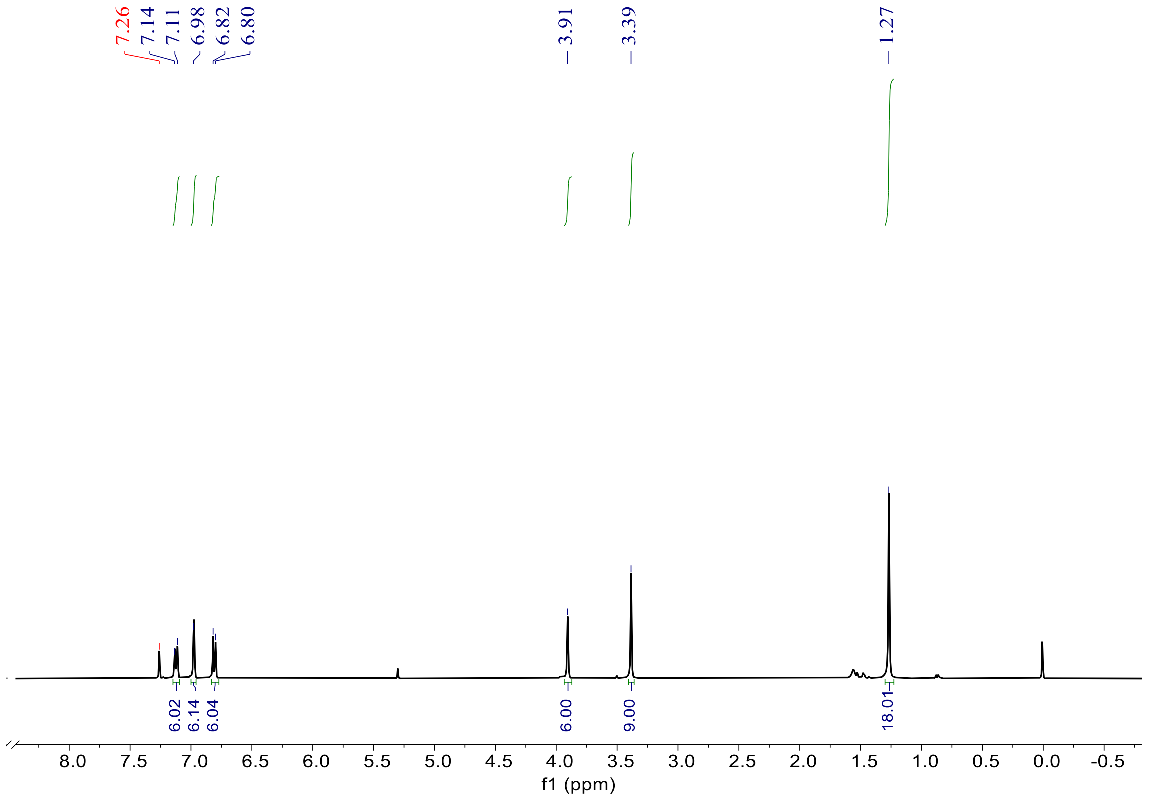

Supplementary Fig. 1. ^{1}H NMR spectrum (400 MHz, $CDCl_3$, 298K) of C[3]A.

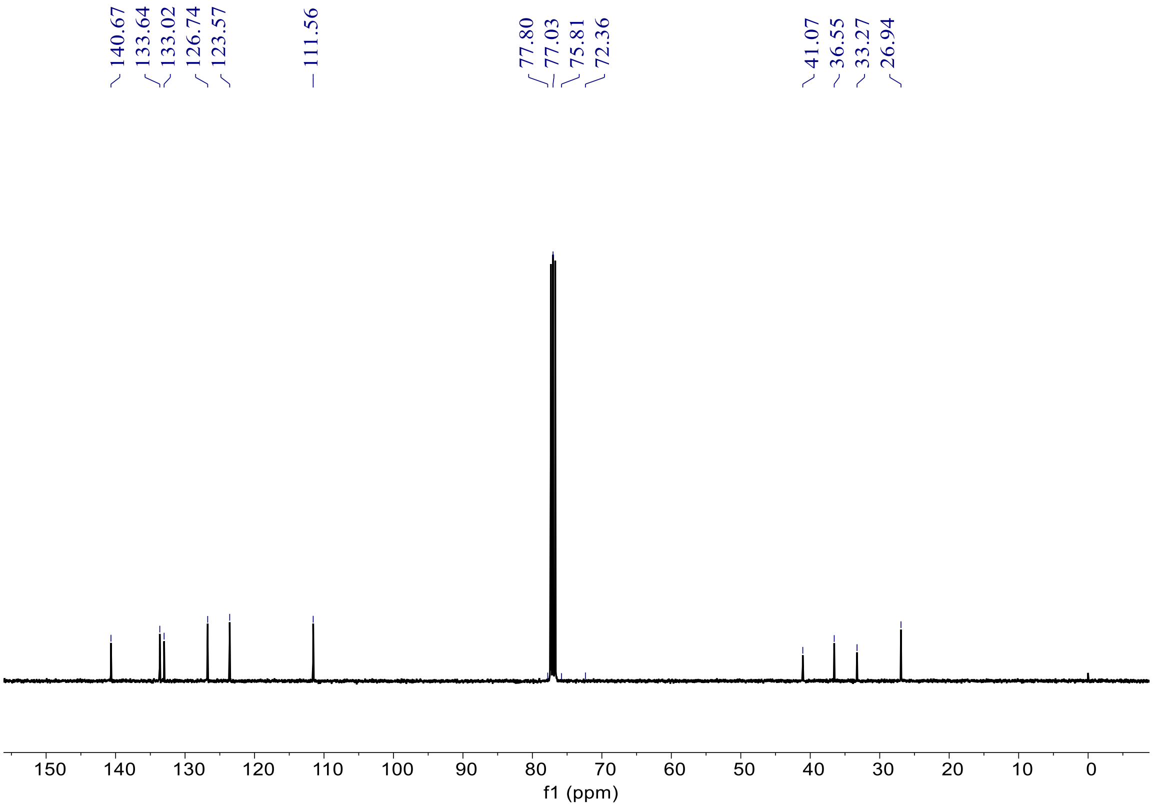

Supplementary Fig. 2. ^{13}C NMR spectrum (101 MHz, $CDCl_3$, 298K) of C[3]A.

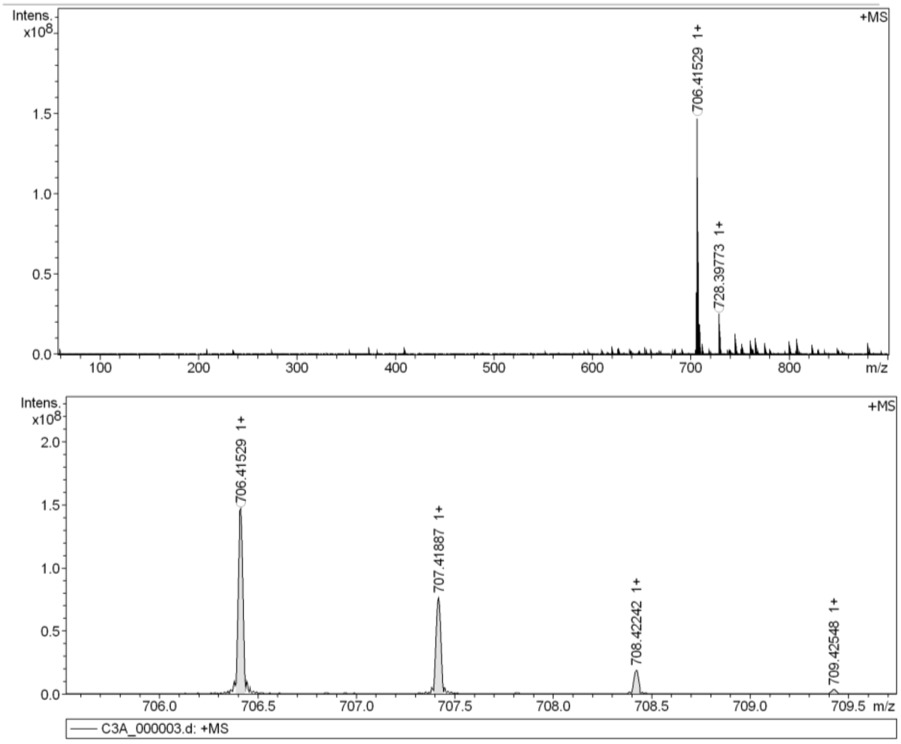

Meas. m/z	#	Ion Formula	Score	m/z	err [ppm]	Mean err [ppm]	mSigma	rdb	e⁻ Conf	N-Rule
706.415289	1	C51H52N3	100.00	706.415575	-0.4	0.2	26.1	27.5	even	ok
728.397728	1	C51H51N3Na	100.00	728.397519	-0.3	-0.3	15.1	27.5	even	ok

Supplementary Fig. 3. HR-MS (ESI) spectrum of C[3]A.

1.3 Preparation of Cocrystals

Preparation of cocrystal G1@C[3]A. 23 mg C[3]A and 3.4 mg G1 were dissolved in dichloromethane (3 mL), then the solution was filtered by a 0.22-μm syringe. With slow vapor diffusion of *n*-hexane into the solution at room temperature for 2~5 days, the deep blue fluorescence parallelogram-shaped G1@C[3]A cocrystals were obtained.

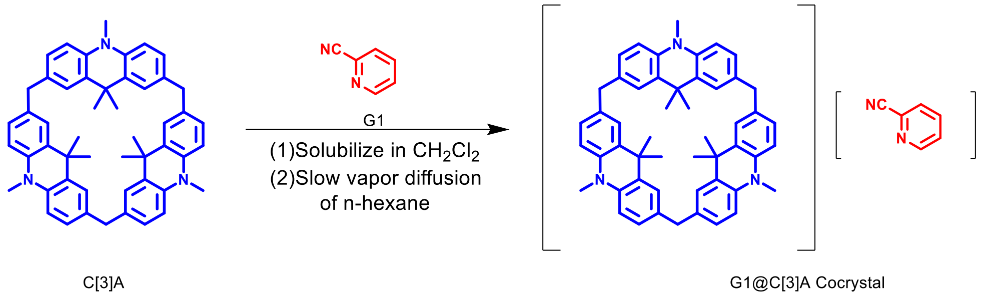

Supplementary Fig. 4. Preparation of cocrystal G1@C[3]A.

Preparation of cocrystal G2@C[3]A. 38 mg C[3]A and 7.0 mg G2 were dissolved in dichloromethane (3.5 mL), then the solution was filtered by a 0.22-μm syringe. With slow vapor diffusion of *n*-hexane into the solution as slowly as possible at 3~5 °C for 2~5 days, the blue fluorescence cuboid-shaped G2@C[3]A cocrystals were obtained.

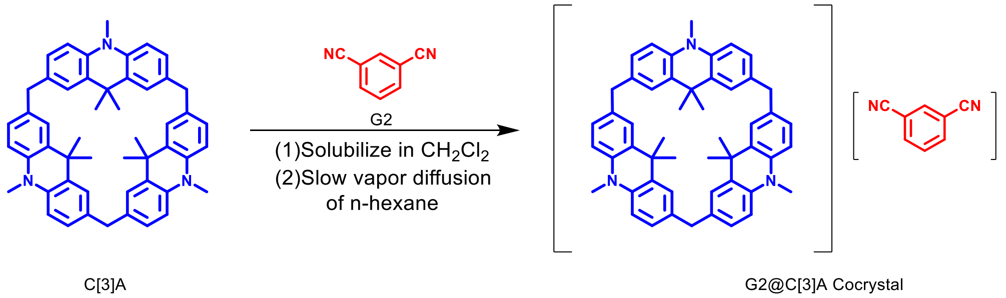

Supplementary Fig. 5. Preparation of cocrystal G2@C[3]A.

Preparation of cocrystal G3@C[3]A. 34 mg C[3]A and 6.3 mg G3 were dissolved in dichloromethane (3.5 mL), then the solution was filtered by a 0.22-μm syringe. With slow vapor diffusion of *n*-hexane into the solution at room temperature for 2~5 days, the blue-green fluorescence parallelogram-shaped G3@C[3]A cocrystals were obtained.

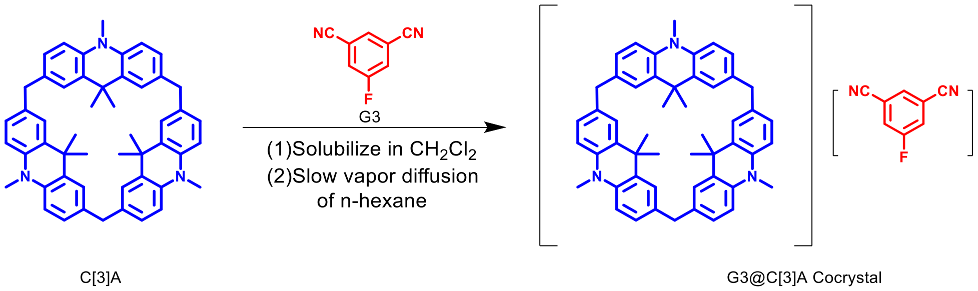

Supplementary Fig. 6. Preparation of cocrystal G3@C[3]A.

Preparation of cocrystal G4@C[3]A. 35 mg C[3]A and 7.3 mg G4 were dissolved in dichloromethane (3.5 mL), then the solution was filtered by a 0.22-μm syringe. With slow vapor diffusion of *n*-hexane into the solution at room temperature for 2~5 days, the green fluorescence cuboid-shaped G4@C[3]A cocrystals were obtained.

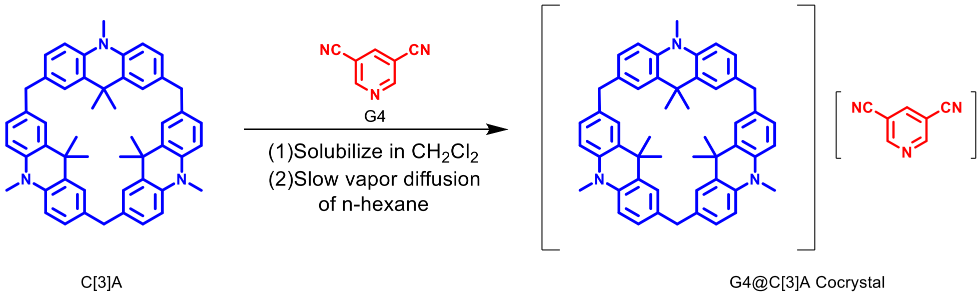

Supplementary Fig. 7. Preparation of cocrystal G4@C[3]A.

Preparation of cocrystal G5@C[3]A. 30 mg C[3]A and 6.5 mg G5 were dissolved in *n*-hexane/dichloromethane (3 mL / 3 mL), then the solution was filtered by a 0.22-μm syringe. The obtained homogenous solution slowly evaporates at room temperature for 2~5 days, and the chartreuse fluorescence parallelogram-shaped G5@C[3]A cocrystals were obtained.

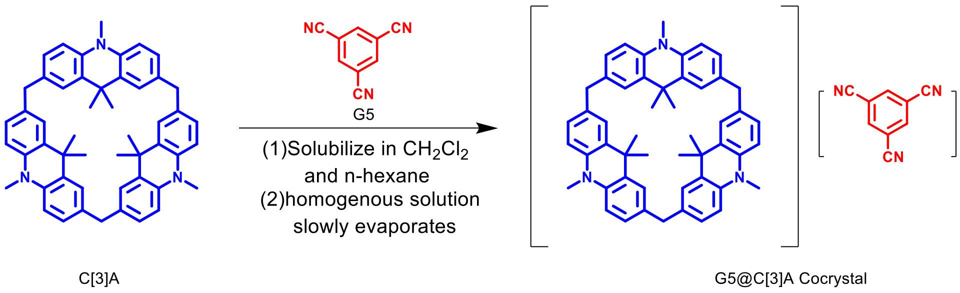

Supplementary Fig. 8. Preparation of cocrystal G5@C[3]A.

Preparation of cocrystal G6@C[3]A. 36 mg C[3]A and 6.6 mg G6 were dissolved in dichloromethane (3.5 mL), then the solution was filtered by a 0.22-μm syringe. With slow vapor diffusion of *n*-hexane into the solution as slowly as possible at 3~5 °C for 2~5 days, the orange fluorescence G6@C[3]A cubic cocrystals were obtained.

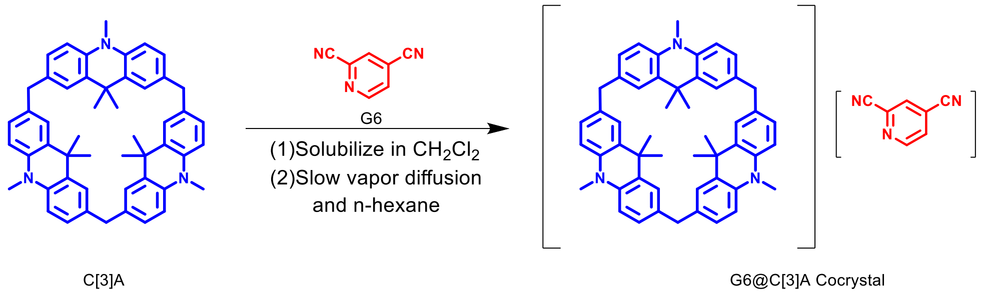

Supplementary Fig. 9. Preparation of cocrystal G6@C[3]A.

Preparation of cocrystal G7@C[3]A. 34 mg C[3]A and 6.3 mg G7 were dissolved in dichloromethane (4.0 mL), then the solution was filtered by a 0.22-μm syringe. With slow vapor diffusion of *n*-hexane into the solution as slowly as possible at 3~5 °C for 5~8 days, the red fluorescence block-shaped G7@C[3]A cocrystals were obtained.

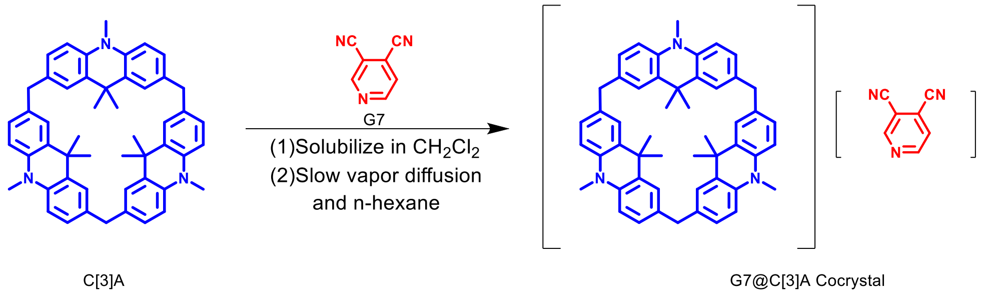

Supplementary Fig. 10. Preparation of cocrystal G7@C[3]A.

2. Supplementary Discussion

2.1 Frontier Orbital Calculations of G1~G7

Supplementary Table 1. HOMO and LUMO distribution and frontier orbital energy levels calculation results of G1~G7.

Guest	Chemical structure	Molecular orbital		HOMO [eV]	LUMO [eV]
		HOMO	LUMO		
2-cyanopyridine (G1)	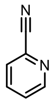	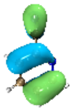	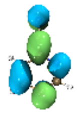	-7.64	-1.83
1,3-dicyanobenzene (G2)	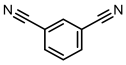	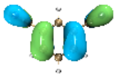	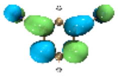	-7.83	-2.24
3,5-dicyanofluorobenzene (G3)	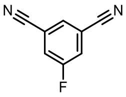	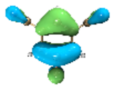	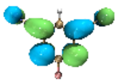	-7.98	-2.51
Pyridine-3,5-dicarbonitrile (G4)	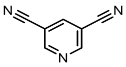	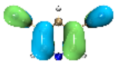	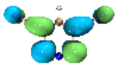	-8.19	-2.59
1,3,5-benzenetricarbonitrile (G5)	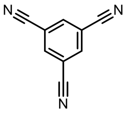	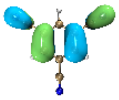	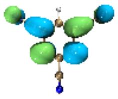	-8.43	-2.83
2,4-pyridinedicarbonitrile (G6)	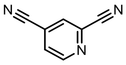	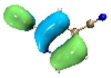	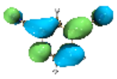	-8.28	-2.84
3,4-dicyanopyridine (G7)	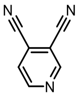	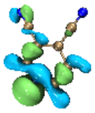	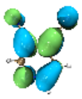	-8.22	-2.91

2.2 Crystal Structures and Crystal Data

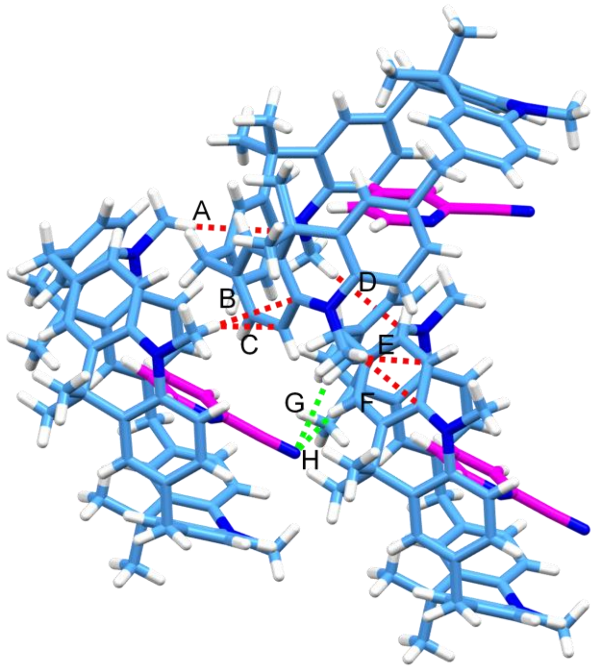

Supplementary Fig. 11. The interactions between the adjacent C[3]A macrocycles including C–H···π interactions (red dash lines A, B, C, D, E and F, with the distances of 2.81 Å, 2.87 Å, 2.82 Å, 2.81 Å, 2.82 Å, and 2.87 Å, respectively). The cyano group of the encapsulated G1 molecules further interacts with the adjacent C[3]A through C–H···N hydrogen bond interactions (green dash lines G and H, with the distances of 2.71 Å and 2.74 Å, respectively).

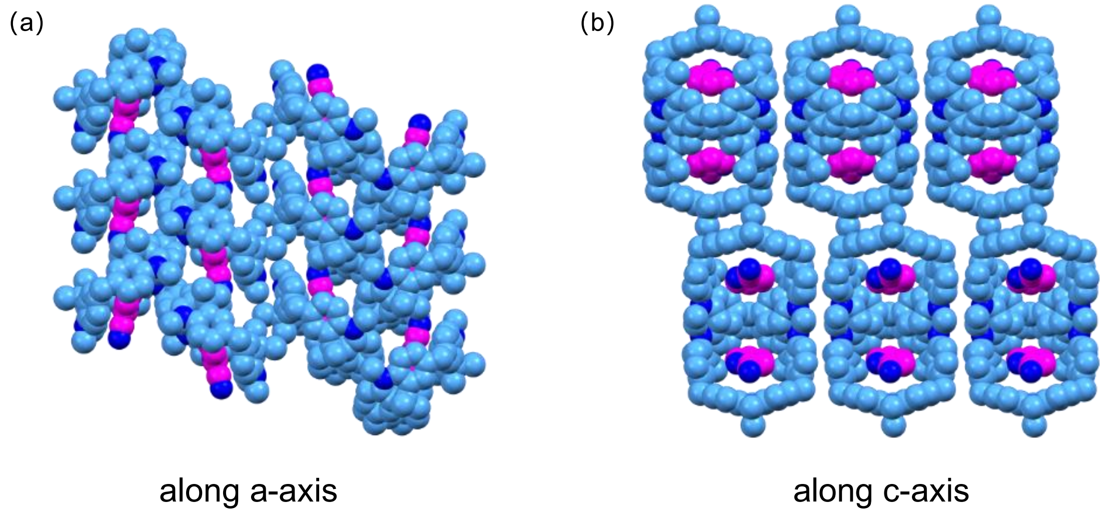

Supplementary Fig. 12. Packing mode of G1@C[3]A along *a*-axis (a) and *c*-axis (b). Partial hydrogen atoms and the solvents were omitted for clarity.

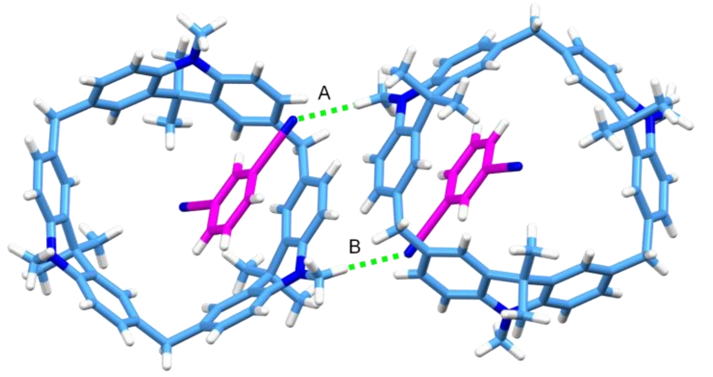

Supplementary Fig. 13. The cyano group of the encapsulated G2 molecules further interacts with the adjacent C[3]A macrocycles through C−H···N hydrogen bond interactions (green dash lines A and B, with the distances of 2.64 Å and 2.64 Å, respectively).

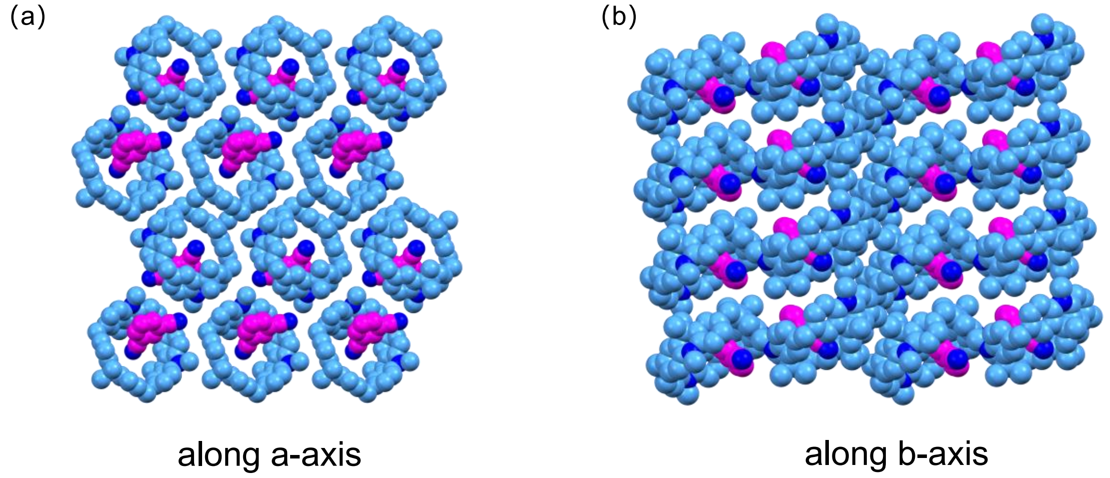

Supplementary Fig. 14. Packing mode of G2@C[3]A along *a*-axis (a) and *b*-axis (b). Partial hydrogen atoms and the solvents were omitted for clarity.

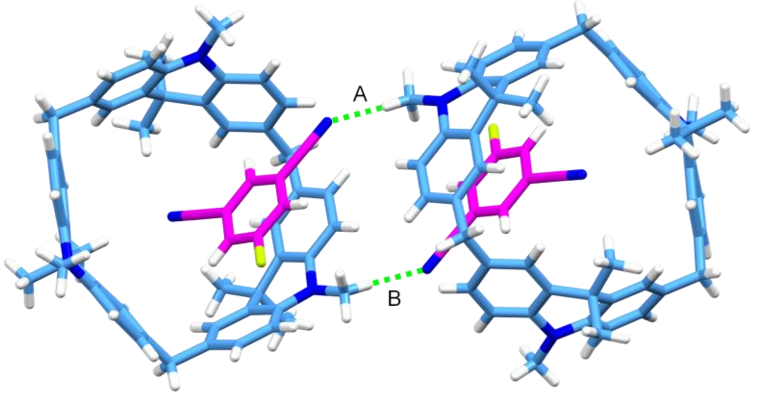

Supplementary Fig. 15. The cyano group of the encapsulated G3 molecules further interacts with the adjacent C[3]A macrocycles through C−H···N hydrogen bond interactions (green dash lines A and B, with the distances of 2.71 Å and 2.71 Å, respectively).

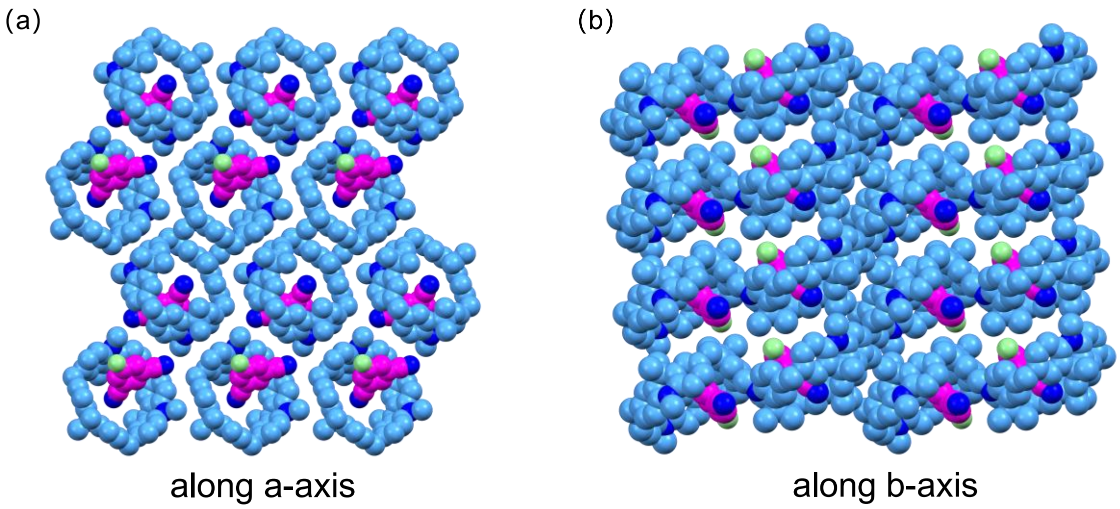

Supplementary Fig. 16. Packing mode of G3@C[3]A along *a*-axis (a) and *b*-axis (b). Partial hydrogen atoms and the solvents were omitted for clarity.

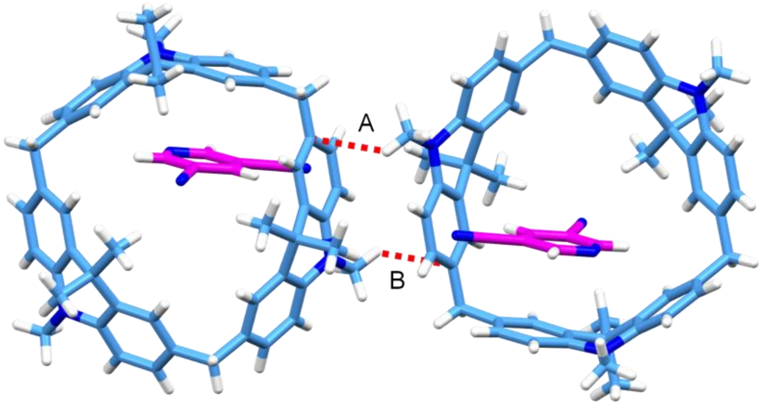

Supplementary Fig. 17. The interactions between two adjacent C[3]A macrocycles including C–H···π interactions (red dash lines A and B, with the distances of 2.61 Å and 2.61 Å, respectively).

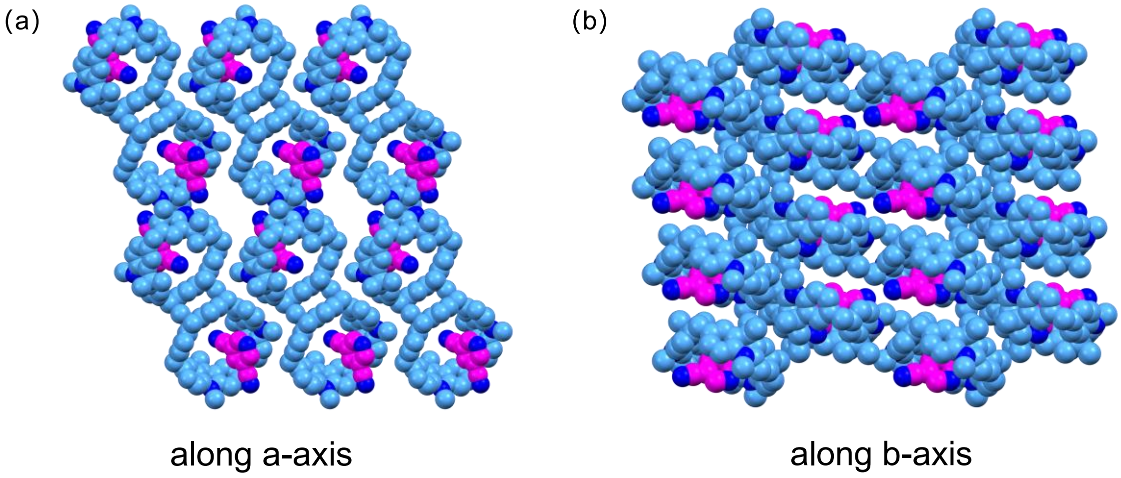

Supplementary Fig. 18. Packing mode of G4@C[3]A along *a*-axis (a) and *b*-axis (b). Partial hydrogen atoms and the solvents were omitted for clarity.

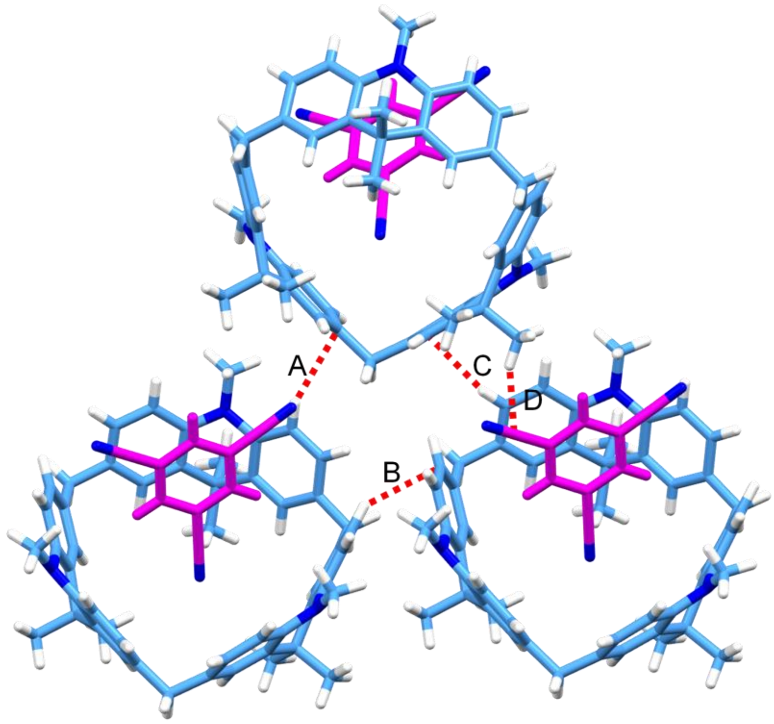

Supplementary Fig. 19. The cyano group of the encapsulated G5 molecules interacts with the adjacent C[3]A macrocycles through C−H···π interactions (red dash lines D, with the distances of 2.79 Å). In addition, the interactions between three adjacent C[3]A macrocycles including C−H···π interactions (red dash lines A, B and C, with the distances of 2.88 Å, 2.88 Å and 2.89 Å, respectively).

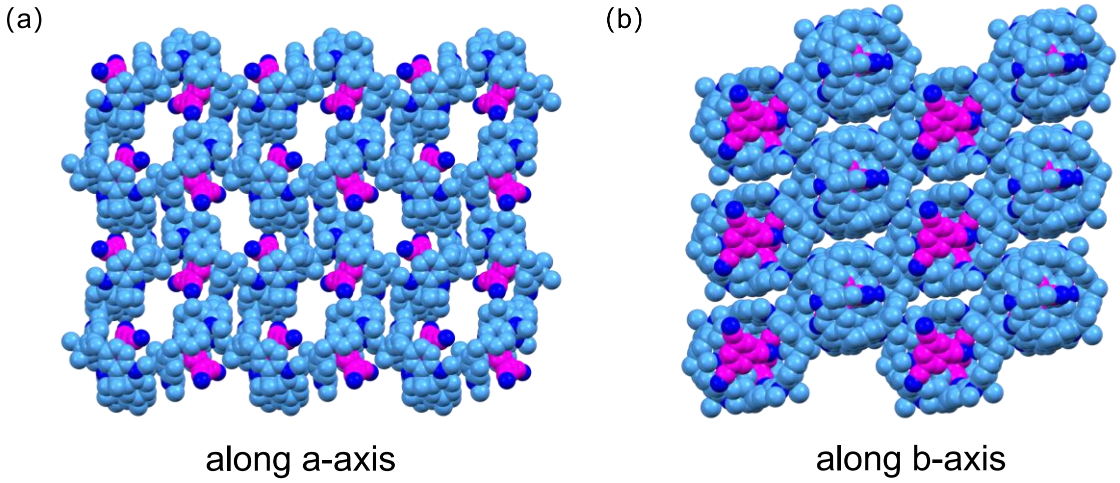

Supplementary Fig. 20. Packing mode of G5@C[3]A along *a*-axis (a) and *b*-axis (b). Partial hydrogen atoms and the solvents were omitted for clarity.

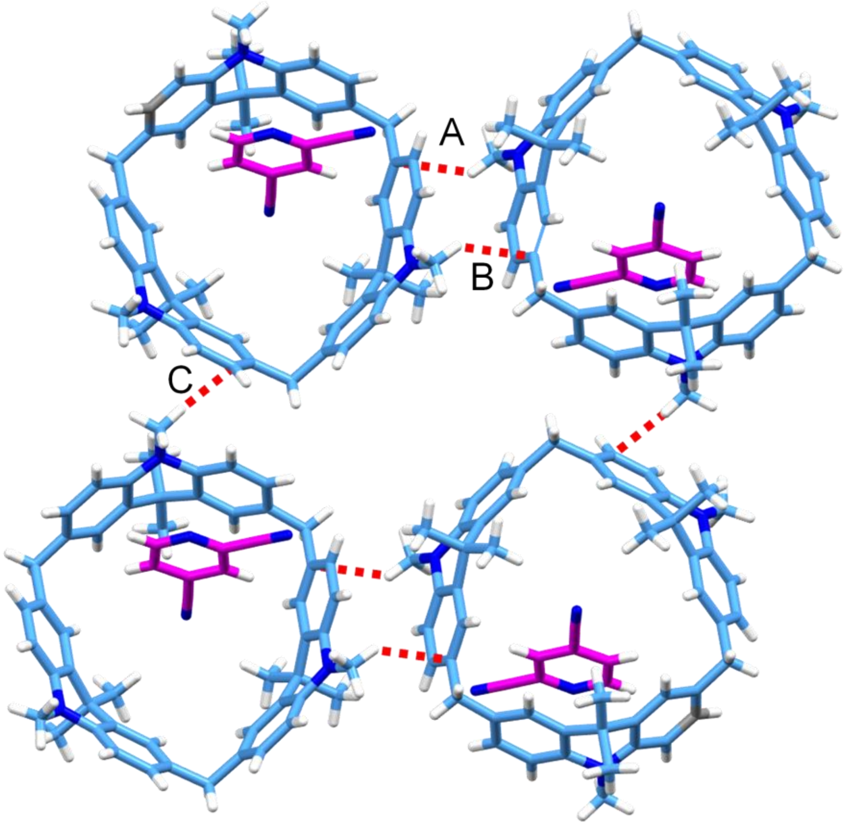

Supplementary Fig. 21. The interactions between adjacent C[3]A macrocycles including C–H···π interactions (red dash lines A, B and C, with the distances of 2.82 Å, 2.82 Å and 2.90 Å, respectively).

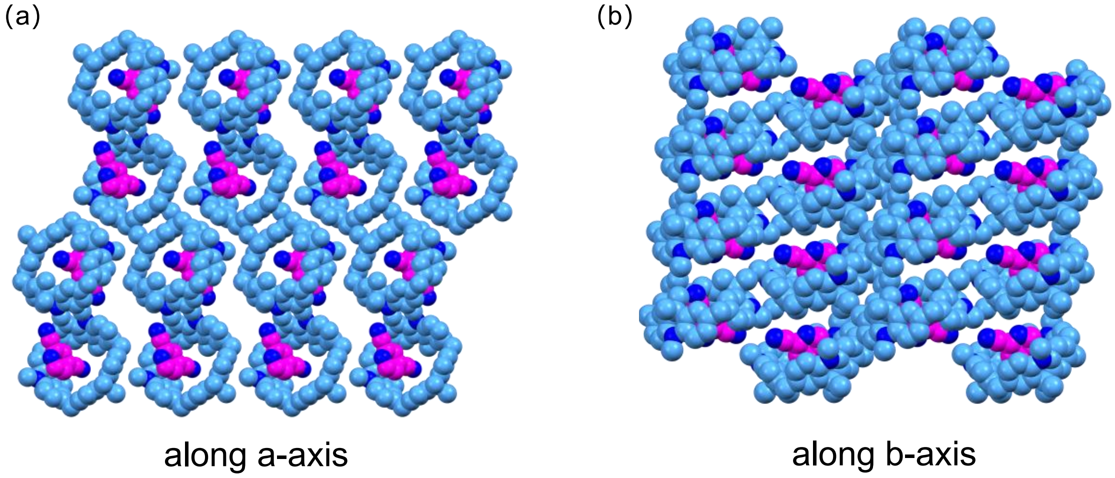

Supplementary Fig. 22. Packing mode of G6@C[3]A along *a*-axis (a) and *b*-axis (b). Partial hydrogen atoms and the solvents were omitted for clarity.

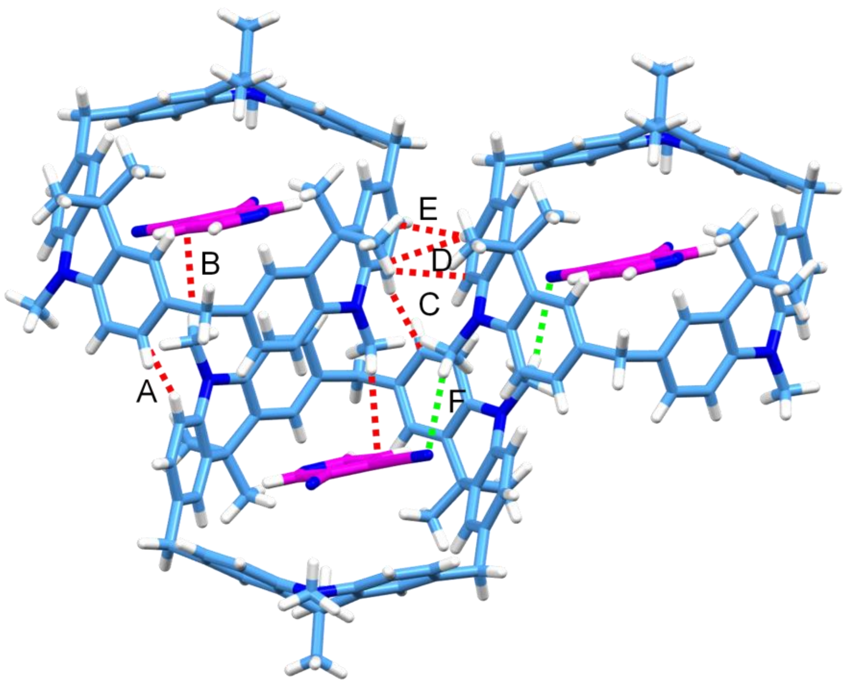

Supplementary Fig. 23. The aromatic ring of the encapsulated G7 molecules interacts with the adjacent C[3]A macrocycles through C−H···π interactions (red dash lines B, with the distances of 2.79 Å). In addition, the interactions between three adjacent C[3]A macrocycles including C−H···π interactions (red dash lines A, B and C, with the distances of 2.88 Å, 2.88 Å and 2.89 Å, respectively). The cyano group of the encapsulated G7 molecules interacts with the adjacent C[3]A macrocycles through C−H···N hydrogen bond interactions (green dash lines F, with the distances of 2.68 Å).

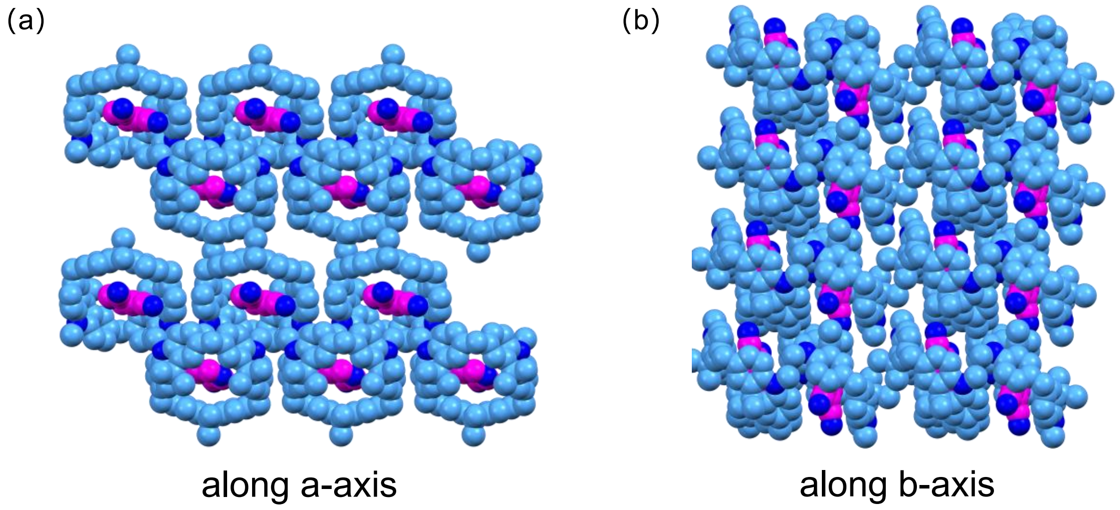

Supplementary Fig. 24. Packing mode of G7@C[3]A along *a*-axis (a) and *b*-axis (b). Partial hydrogen atoms and the solvents were omitted for clarity.

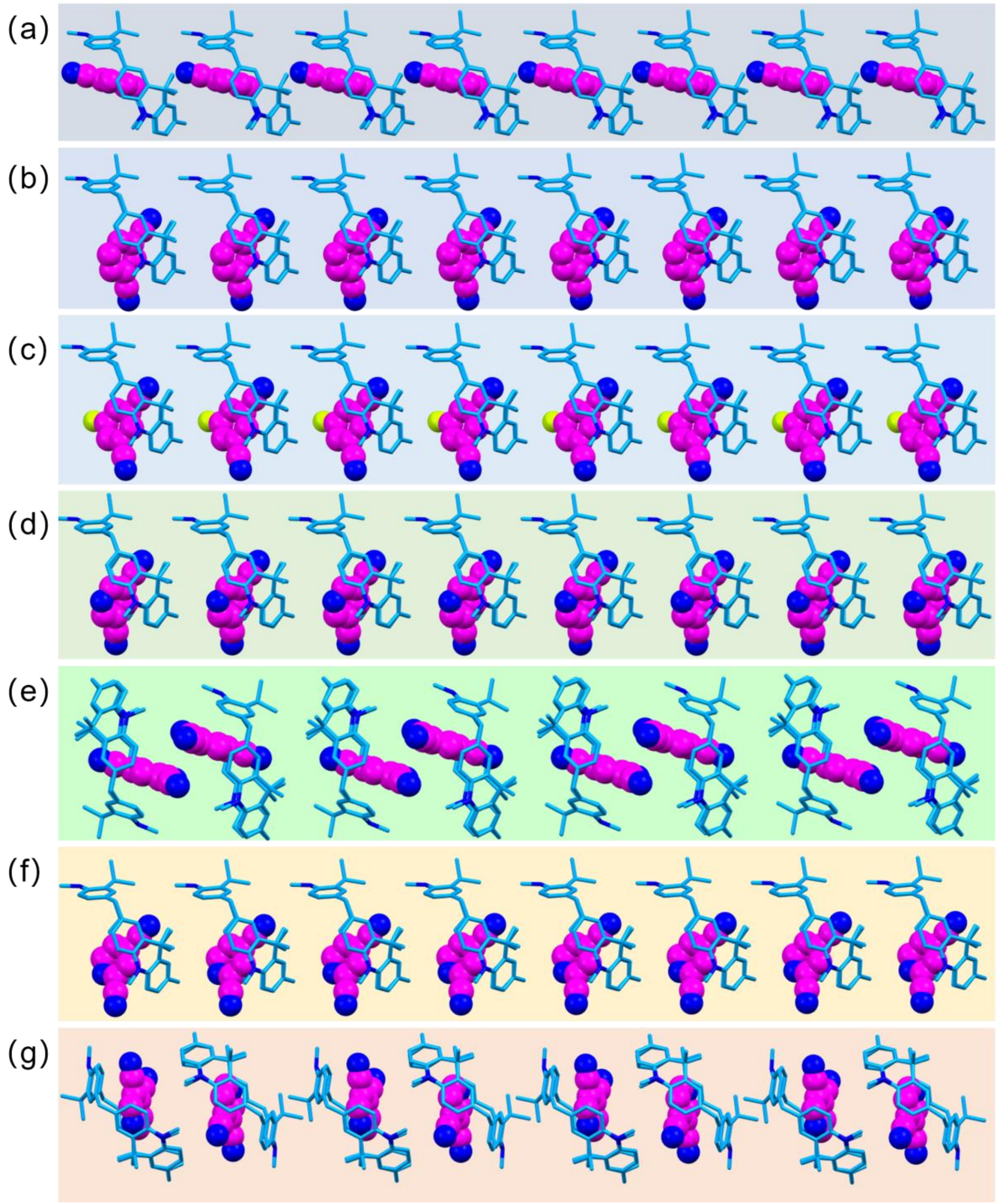

Supplementary Fig. 25. Crystal packing mode of (a) G1@C[3]A, (b) G2@C[3]A, (c) G3@C[3]A, (d) G4@C[3]A, (e) G5@C[3]A, (f) G6@C[3]A and (g) G7@C[3]A, showing that the host−guest complexes formed by C[3]A and the guests stack to form 1D linear superstructures.

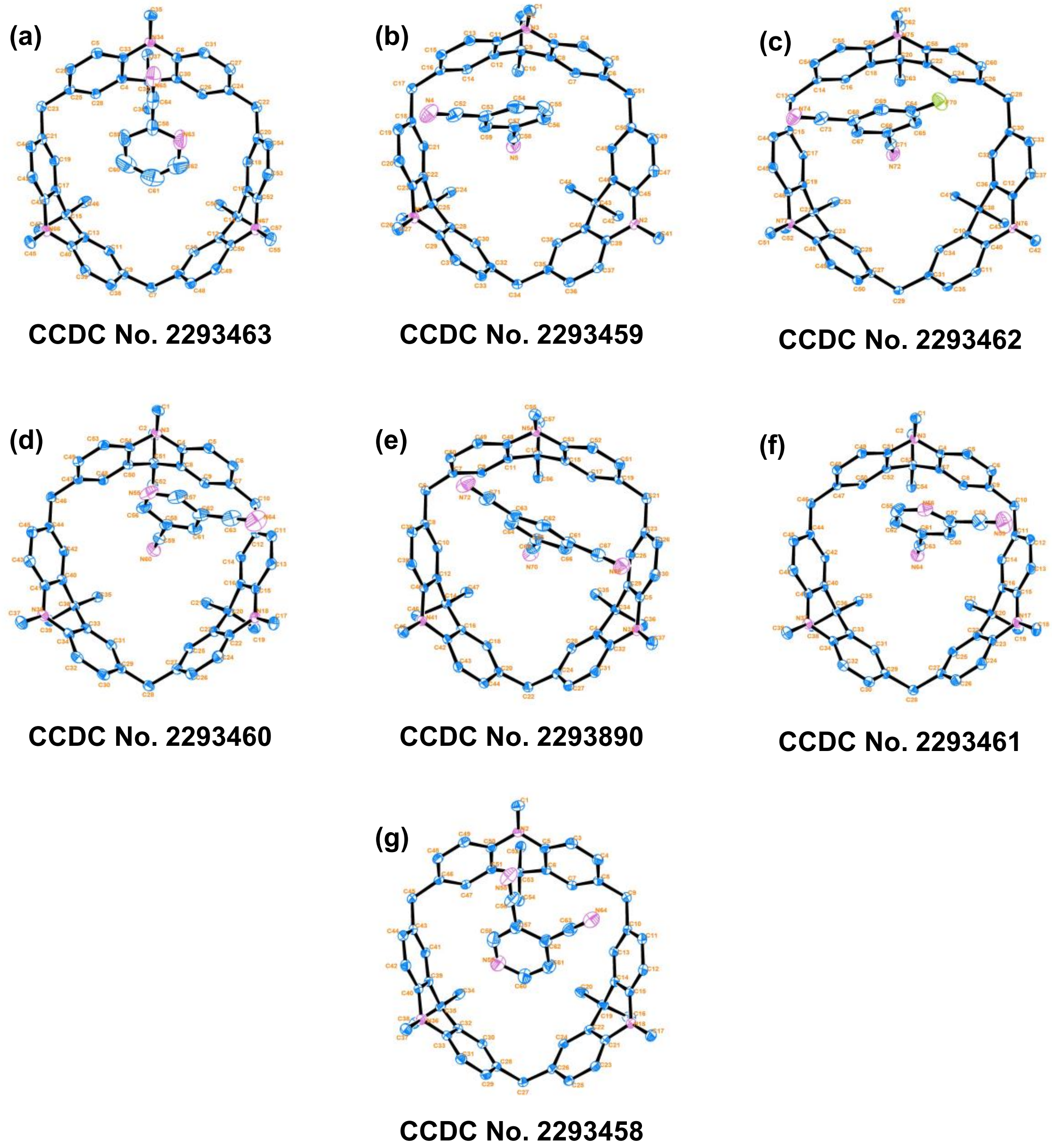

Supplementary Fig. 26. ORTEP-3 style illustration[14] of crystal structure (a) G1@C[3]A, (b) G2@C[3]A, (c) G3@C[3]A, (d) G4@C[3]A, (e) G5@C[3]A, (f) G6@C[3]A and (g) G7@C[3]A. Thermal ellipsoids are set 30% probability level. All the hydrogen atoms are omitted for clarity.

Supplementary Table 2. Crystal data and structure refinement parameters for G1@C[3]A.

**Identification code**	G1@C[3]A
**CCDC No.**	2293463
**Empirical formula**	$C_{57}H_{55}N_5$
**Formula weight**	810.06
**Temperature/K**	169.99(10)
**Crystal system**	monoclinic
**Space group**	$P2_1/c$
**a/Å**	13.45693(19)
**b/Å**	37.4717(6)
**c/Å**	9.52924(13)
**α/°**	90
**β/°**	99.4093(13)
**γ/°**	90
**Volume/Å^3**	4740.50(12)
**Z**	4
**ρ_{calc}g/cm^3**	1.135
**μ/mm^{-1}**	0.508
**F(000)**	1728.0
**Crystal size/mm^3**	0.5 × 0.2 × 0.2
**Radiation**	Cu Kα (λ = 1.54184)
**2Θ range for data collection/°**	4.716 to 151.754
**Reflections collected**	42605
**Independent reflections**	9580 [R_{int} = 0.0449, R_{sigma} = 0.0343]
**Data/restraints/parameters**	9580/0/569
**Goodness-of-fit on F^2**	1.038
**Final R indexes [I>=2σ (I)]**	R_1 = 0.0596, wR_2 = 0.1633
**Final R indexes [all data]**	R_1 = 0.0660, wR_2 = 0.1677
**Largest diff. peak/hole / e Å^{-3}**	0.53/-0.29
**Crystallization solvents**	Hexane/dichloromethane

Supplementary Table 3. Crystal data and structure refinement parameters for G2@C[3]A.

Identification code	G2@C[3]A
CCDC No.	2293459
Empirical formula	$C_{59}H_{55}N_5$
Formula weight	834.08
Temperature/K	169.99(10)
Crystal system	triclinic
Space group	*P*-1
a/Å	9.2622(5)
b/Å	13.8172(9)
c/Å	22.2911(7)
α/°	84.062(4)
β/°	84.824(4)
γ/°	77.376(5)
Volume/Å^3	2762.1(3)
Z	2
ρcalcg/cm^3	1.003
μ/mm^{-1}	0.449
F(000)	888.0
Crystal size/mm^3	0.6 × 0.22 × 0.2
Radiation	Cu Kα (λ = 1.54184)
2Θ range for data collection/°	6.58 to 153.488
Reflections collected	35886
Independent reflections	11096 [R_{int} = 0.0465, R_{sigma} = 0.0439]
Data/restraints/parameters	11096/0/586
Goodness-of-fit on F^2	1.036
Final R indexes [I>=2σ (I)]	R_1 = 0.0462, wR_2 = 0.1147
Final R indexes [all data]	R_1 = 0.0543, wR_2 = 0.1188
Largest diff. peak/hole / e Å^{-3}	0.30/-0.21
Crystallization solvents	Hexane/dichloromethane

Supplementary Table 4. Crystal data and structure refinement parameters for G3@C[3]A.

Identification code	G3@C[3]A
CCDC No.	2293462
Empirical formula	$C_{59}H_{54}FN_5$
Formula weight	852.07
Temperature/K	169.99(10)
Crystal system	triclinic
Space group	*P*-1
a/Å	9.31340(10)
b/Å	13.8352(2)
c/Å	22.1402(2)
α/°	84.1820(10)
β/°	85.0620(10)
γ/°	77.0460(10)
Volume/Å^3	2759.99(6)
Z	2
ρ_{calc}g/cm^3	1.025
μ/mm^{-1}	0.486
F(000)	904.0
Crystal size/mm^3	0.6 × 0.3 × 0.2
Radiation	Cu Kα (λ = 1.54184)
2Θ range for data collection/°	4.02 to 151.668
Reflections collected	35964
Independent reflections	11064 [R_{int} = 0.0500, R_{sigma} = 0.0424]
Data/restraints/parameters	11064/0/595
Goodness-of-fit on F^2	1.063
Final R indexes [I>=2σ (I)]	R_1 = 0.0597, wR_2 = 0.1536
Final R indexes [all data]	R_1 = 0.0669, wR_2 = 0.1584
Largest diff. peak/hole / e Å^{-3}	0.51/-0.39
Crystallization solvents	Hexane/dichloromethane

Supplementary Table 5. Crystal data and structure refinement parameters for G4@C[3]A.

Identification code	G4@C[3]A
CCDC No.	2293460
Empirical formula	$C_{58}H_{54}N_6$
Formula weight	835.07
Temperature/K	169.99(10)
Crystal system	triclinic
Space group	*P*-1
a/Å	9.18700(10)
b/Å	14.0397(2)
c/Å	23.6480(4)
α/°	73.070(2)
β/°	79.8290(10)
γ/°	76.1150(10)
Volume/Å^3	2813.97(8)
Z	2
ρcalcg/cm^3	0.986
μ/mm^{-1}	0.447
F(000)	888.0
Crystal size/mm^3	0.3 × 0.2 × 0.1
Radiation	Cu Kα (λ = 1.54184)
2Θ range for data collection/°	6.718 to 151.646
Reflections collected	42894
Independent reflections	11385 [R_{int} = 0.0848, R_{sigma} = 0.0614]
Data/restraints/parameters	11385/0/586
Goodness-of-fit on F^2	1.030
Final R indexes [I>=2σ (I)]	R_1 = 0.0612, wR_2 = 0.1727
Final R indexes [all data]	R_1 = 0.0669, wR_2 = 0.1781
Largest diff. peak/hole / e Å^{-3}	0.38/-0.30
Crystallization solvents	Hexane/dichloromethane

Supplementary Table 6. Crystal data and structure refinement parameters for G5@C[3]A.

Identification code	G5@C[3]A
CCDC No.	2293890
Empirical formula	$C_{60}H_{54}N_6$
Formula weight	855.09
Temperature/K	169.99(10)
Crystal system	monoclinic
Space group	$P2_1/n$
a/Å	13.10913(12)
b/Å	18.7865(2)
c/Å	21.9041(2)
α/°	90
β/°	91.9896(9)
γ/°	90
Volume/Å^3	5391.18(10)
Z	4
ρ_{calc}g/cm^3	1.054
μ/mm^{-1}	0.468
F(000)	1816.0
Crystal size/mm^3	0.1 × 0.08 × 0.3
Radiation	Cu Kα (λ = 1.54184)
2Θ range for data collection/°	6.2 to 151.782
Reflections collected	44295
Independent reflections	10832 [R_{int} = 0.0457, R_{sigma} = 0.0354]
Data/restraints/parameters	10832/0/604
Goodness-of-fit on F^2	1.026
Final R indexes [I>=2σ (I)]	R_1 = 0.0493, wR_2 = 0.1320
Final R indexes [all data]	R_1 = 0.0555, wR_2 = 0.1368
Largest diff. peak/hole / e Å^{-3}	0.35/-0.31
Crystallization solvents	Hexane/dichloromethane

Supplementary Table 7. Crystal data and structure refinement parameters for G6@C[3]A.

Identification code	G6@C[3]A
**CCDC No.**	2293461
**Empirical formula**	$C_{58}H_{54}N_6$
**Formula weight**	835.07
**Temperature/K**	169.99(10)
**Crystal system**	triclinic
**Space group**	*P*-1
**a/Å**	9.1869(2)
**b/Å**	14.0141(2)
**c/Å**	23.3634(3)
**α/°**	106.0480(10)
**β/°**	91.4250(10)
**γ/°**	104.2600(10)
**Volume/Å^3**	2887.67(8)
**Z**	2
**ρ_{calc}g/cm^3**	0.0995
**μ/mm^{-1}**	0.451
**F(000)**	888.0
**Crystal size/mm^3**	0.4 × 0.3 × 0.1
**Radiation**	Cu Kα (λ = 1.54184)
**2Θ range for data collection/°**	6.8 to 151.548
**Reflections collected**	41527
**Independent reflections**	11245 [R_{int} = 0.0837, R_{sigma} = 0.0599]
**Data/restraints/parameters**	11245/0/586
**Goodness-of-fit on F^2**	1.102
**Final R indexes [I>=2σ (I)]**	R_1 = 0.0601, wR_2 = 0.1767
**Final R indexes [all data]**	R_1 = 0.0681, wR_2 = 0.1822
**Largest diff. peak/hole / e Å^{-3}**	0.31/-0.36
**Crystallization solvents**	Hexane/dichloromethane

Supplementary Table 8. Crystal data and structure refinement parameters for G7@C[3]A.

Identification code	G7@C[3]A
**CCDC No.**	2293458
**Empirical formula**	$C_{59}H_{56}Cl_2N_6$
**Formula weight**	919.99
**Temperature/K**	169.99(10)
**Crystal system**	triclinic
**Space group**	*P*-1
**a/Å**	10.0311(2)
**b/Å**	13.2924(3)
**c/Å**	18.3154(4)
**α/°**	90.793(2)
**β/°**	94.824(2)
**γ/°**	98.259(2)
**Volume/Å^3**	2407.41(9)
**Z**	2
**ρ_{calc}g/cm^3**	1.269
**μ/mm^{-1}**	1.567
**F(000)**	972.0
**Crystal size/mm^3**	0.42 × 0.3 × 0.23
**Radiation**	Cu Kα (λ = 1.54184)
**2Θ range for data collection/°**	4.844 to 151.406
**Reflections collected**	30351
**Independent reflections**	9666 [R_{int} = 0.0458, R_{sigma} = 0.0478]
**Data/restraints/parameters**	9666/60/632
**Goodness-of-fit on F^2**	1.068
**Final R indexes [I>=2σ (I)]**	R_1 = 0.0600, wR_2 = 0.1649
**Final R indexes [all data]**	R_1 = 0.0833, wR_2 = 0.1822
**Largest diff. peak/hole / e Å^{-3}**	1.19/-0.40
**Crystallization solvents**	Hexane/dichloromethane

2.3 Powder X-ray Diffraction Analysis

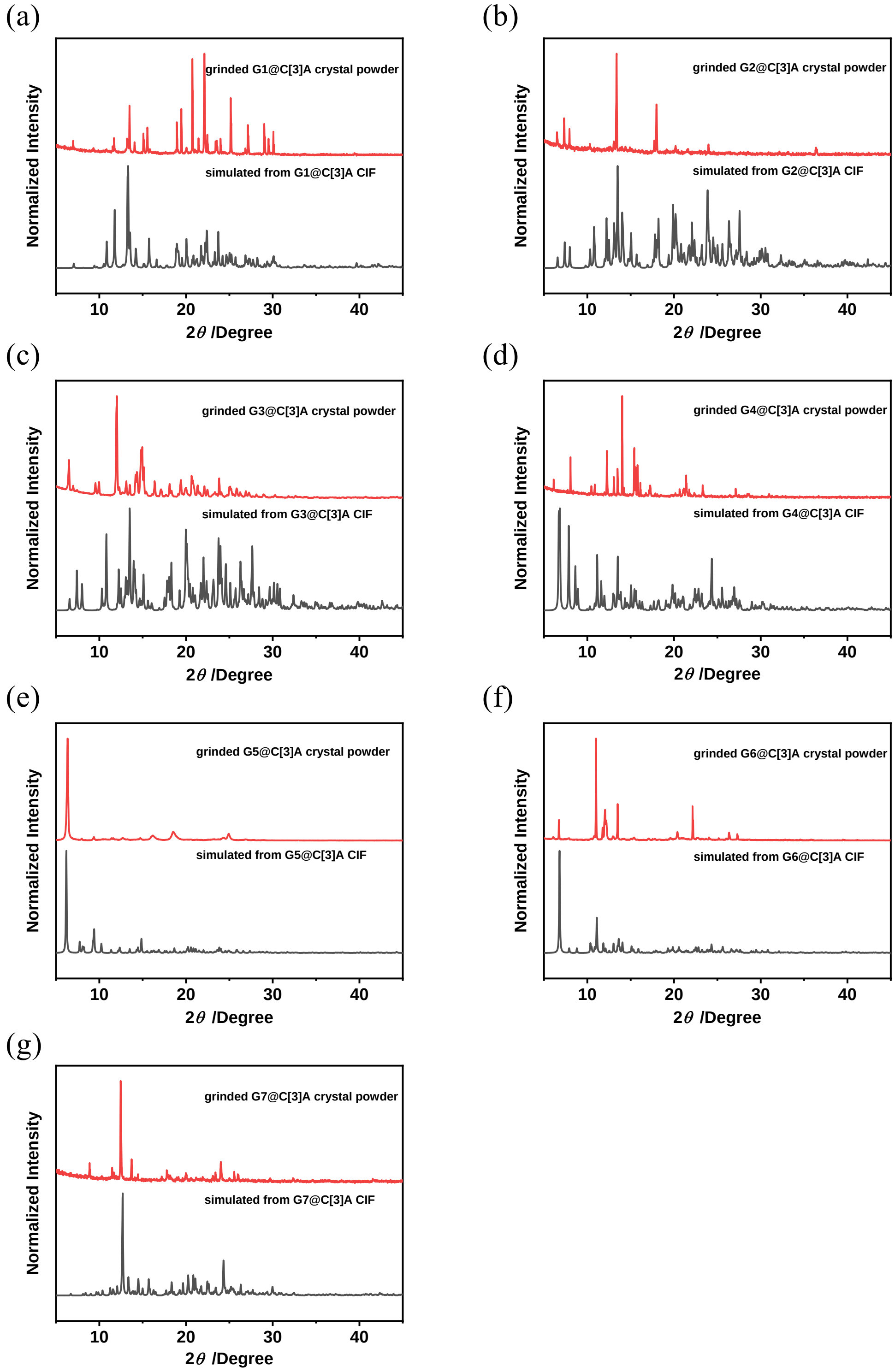

Supplementary Fig. 27. The powder X-ray diffraction (PXRD) patterns (red lines) and corresponding CIF simulation results (dark grey lines) of (a) G1@C[3]A crystal, (b) G2@C[3]A crystal, (c) G3@C[3]A crystal, (d) G4@C[3]A crystal, (e) G5@C[3]A crystal, (f) G6@C[3]A crystal and (g) G7@C[3]A crystal.

2.4 Thermogravimetic Analysis and Differential Scanning Calorimetry

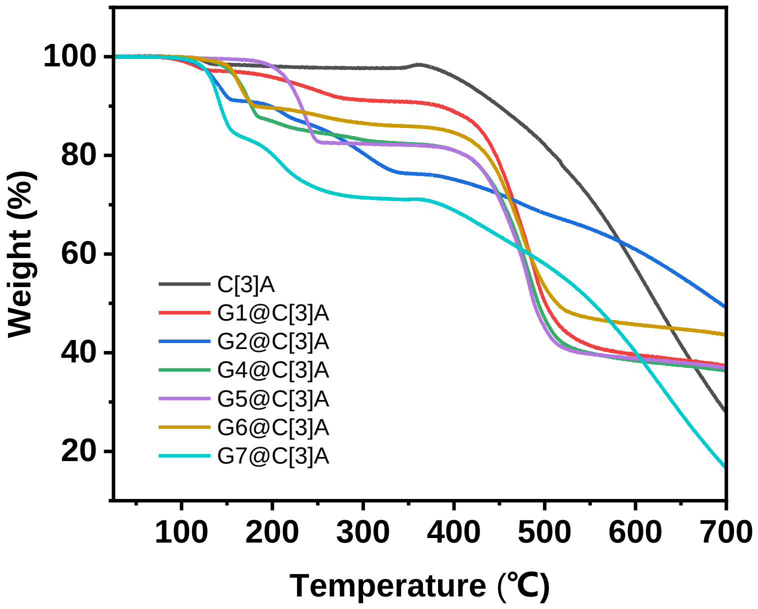

Supplementary Fig. 28. Thermogravimetic analysis of C[3]A and the cocrystals.

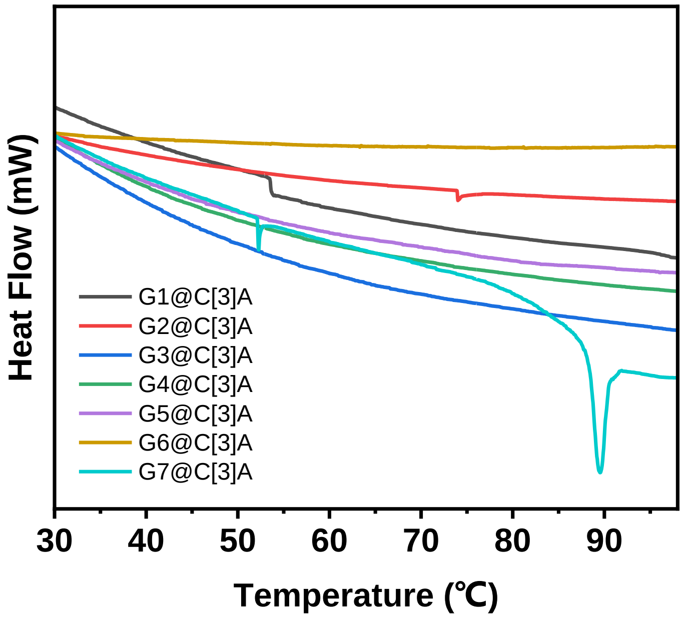

Supplementary Fig. 29. Differential scanning calorimetry of G1@C[3]A~ G7@C[3]A.

2.5 Photophysical Properties

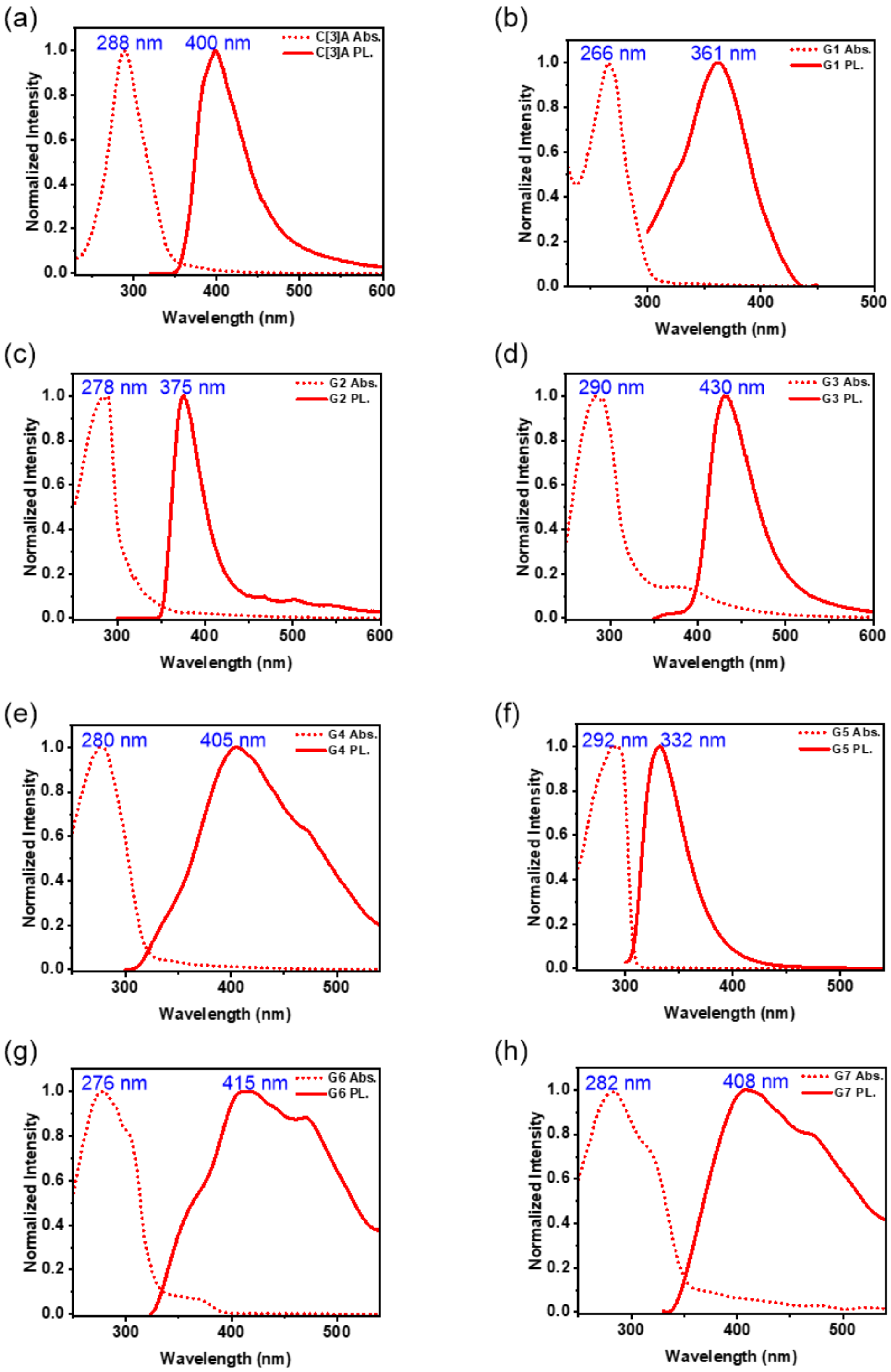

Supplementary Fig. 30. The solid UV-Vis absorption (Abs.) spectra and photoluminescence (PL.) spectra at room temperature: (a) C[3]A, (b) G1, (c) G2, (d) G3, (e) G4, (f) G5, (g) G6 and (h) G7. C[3]A and G2~G7 are tested in solid state, while G1 is tested in toluene solution (10^{-5} mol/L).

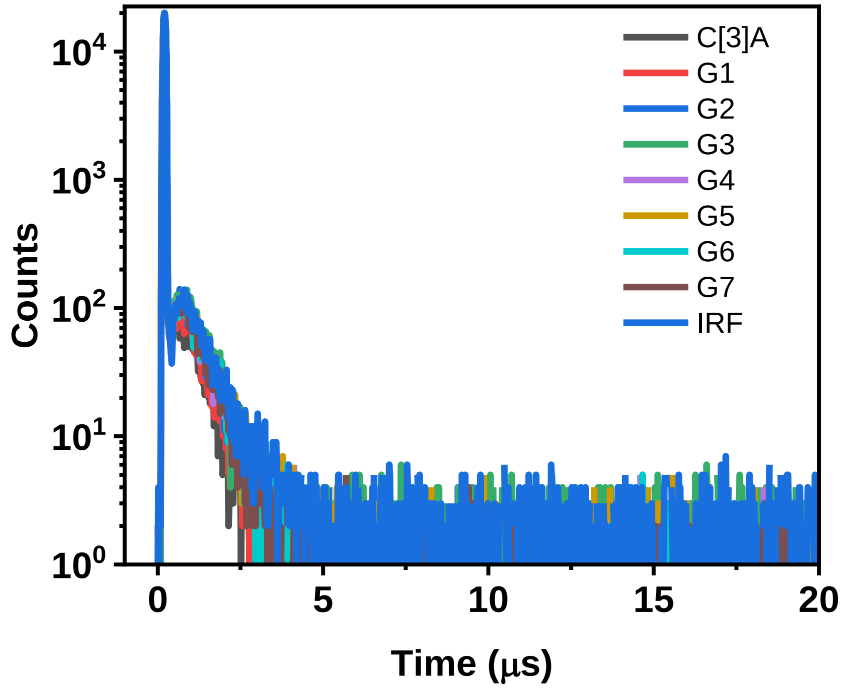

Supplementary Fig. 31. Transient PL decay curves of the host crystal and independent guests at room temperature (IRF, Instrument Response Functions).

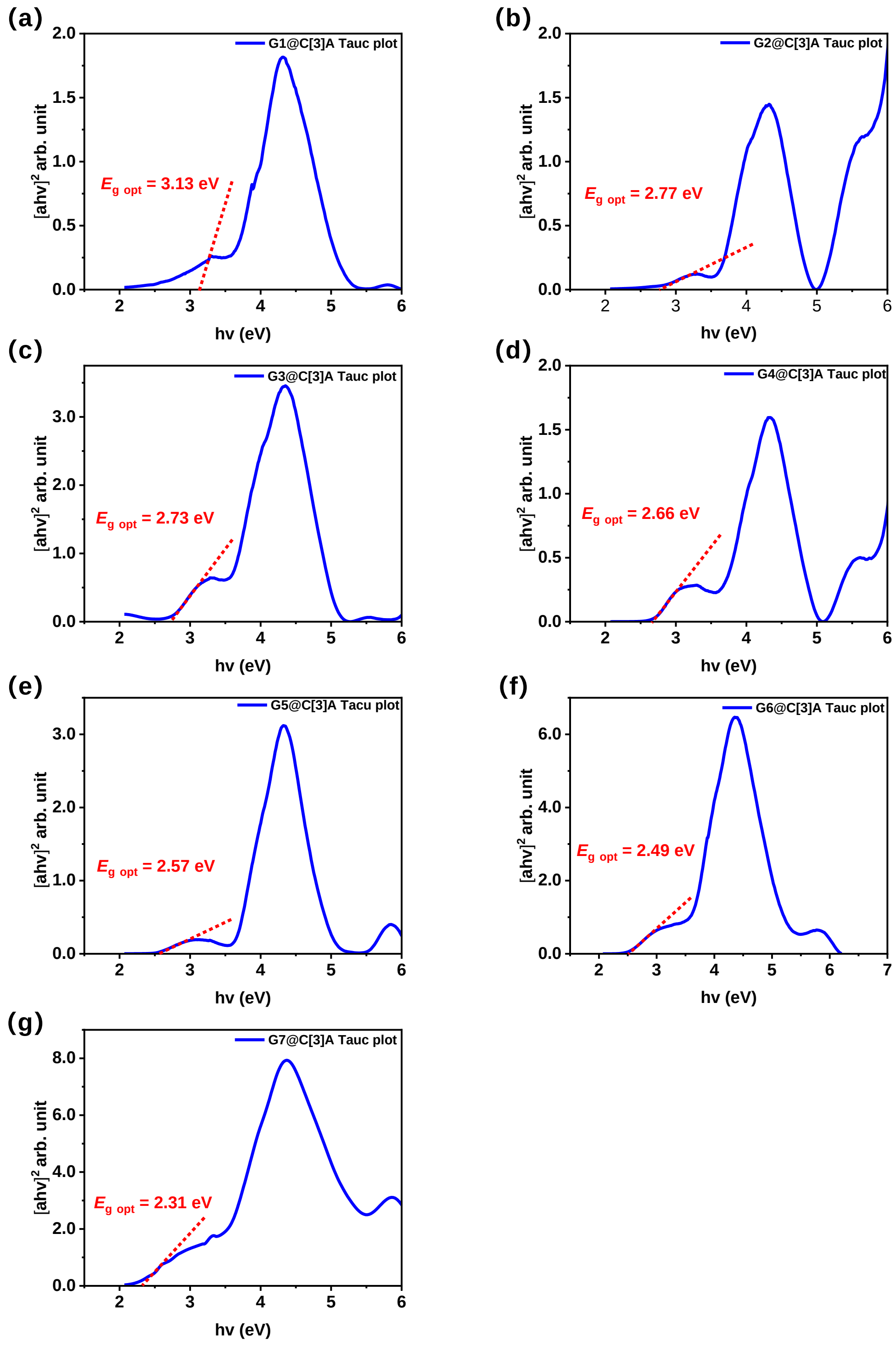

Supplementary Fig. 32. Tauc plot obtained based on UV-Abs. data conversion: (a) G1@C[3]A, (b) G2@C[3]A, (c) G3@C[3]A, (d) G4@C[3]A, (e) G5@C[3]A, (f) G6@C[3]A, (g) G7@C[3]A.

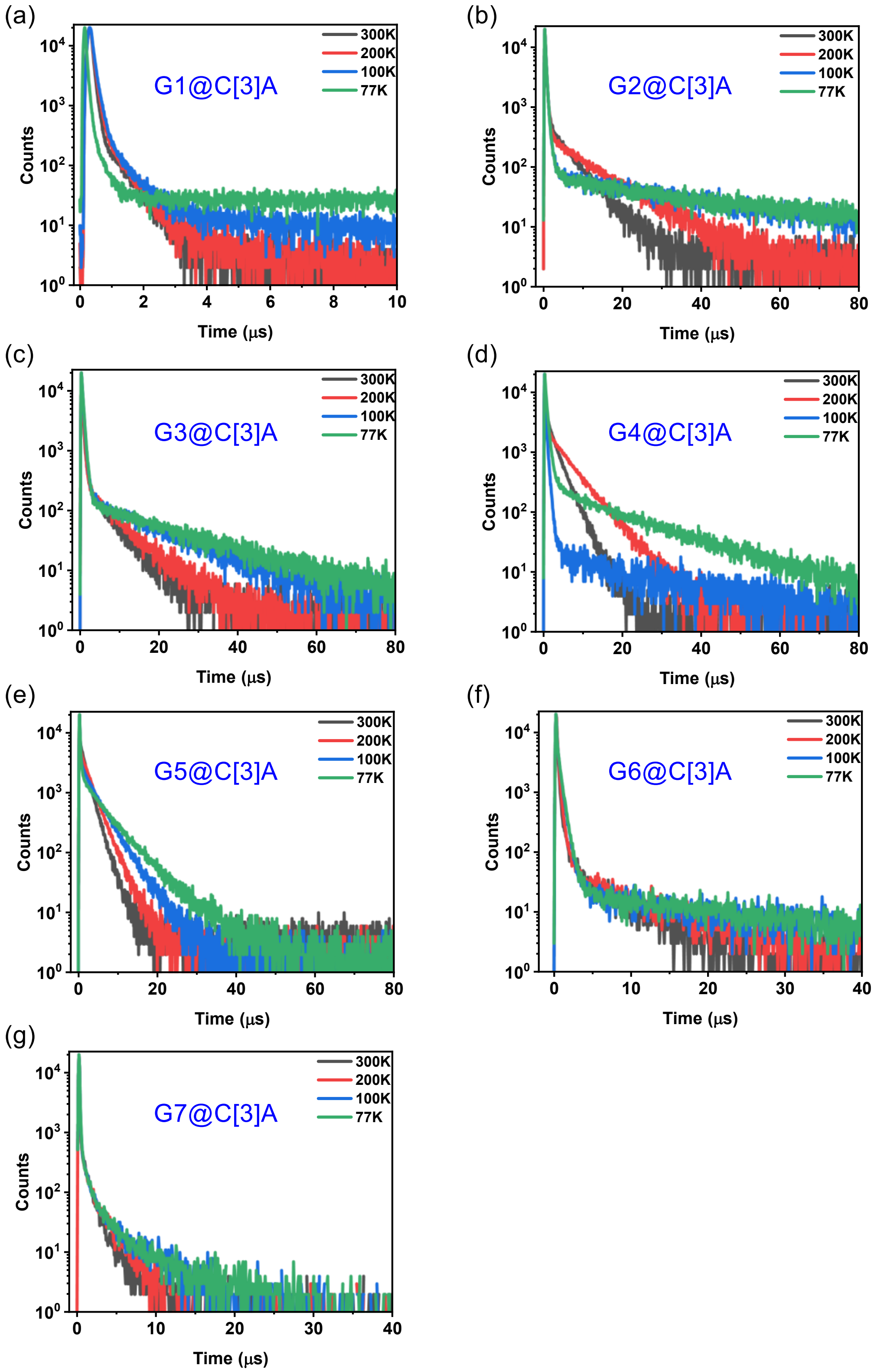

Supplementary Fig. 33. Temperature-dependent transient PL decay curves: (a) G1@C[3]A, (b) G2@C[3]A, (c) G3@C[3]A, (d) G4@C[3]A, (e) G5@C[3]A, (f) G6@C[3]A, (g) G7@C[3]A.

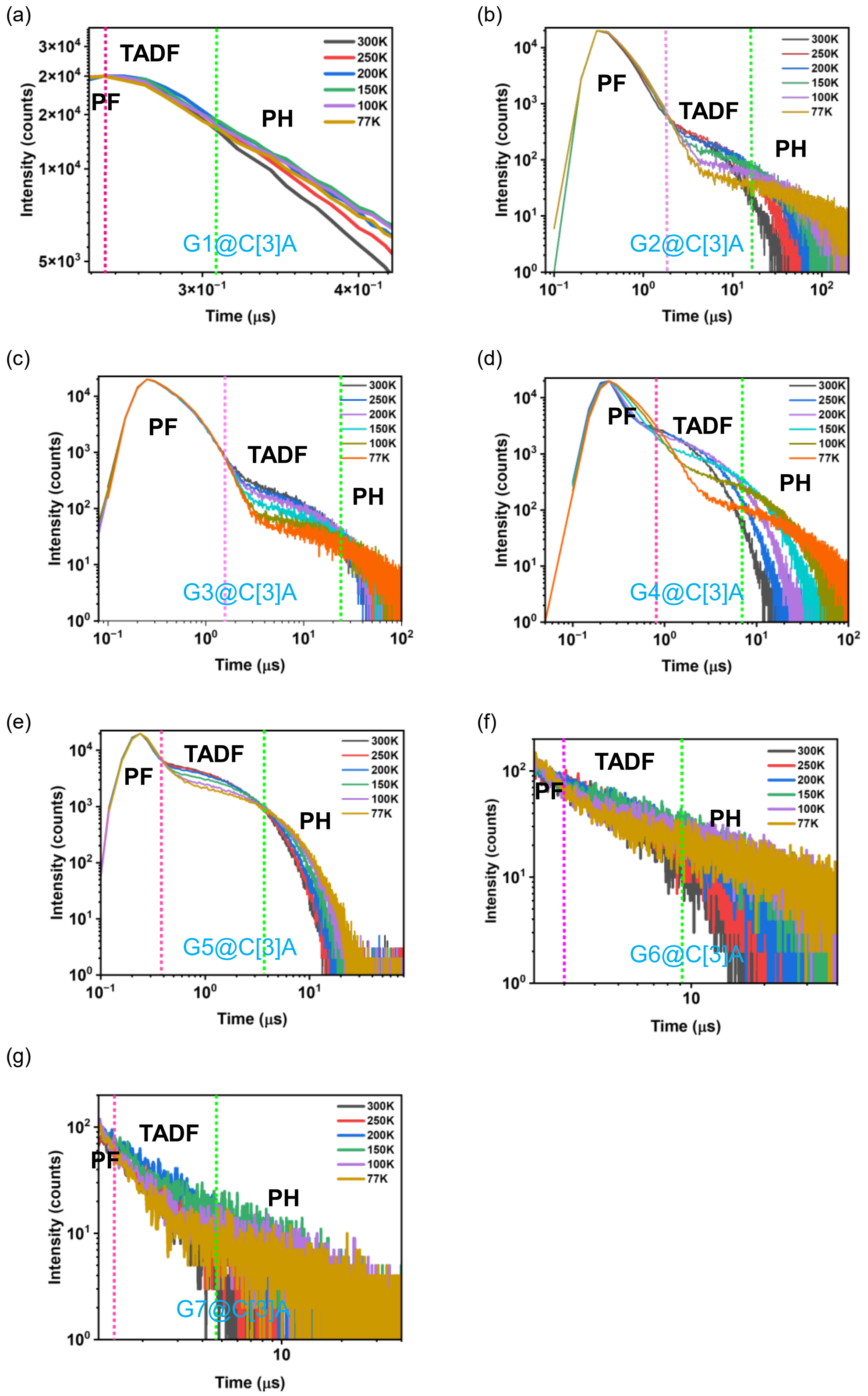

Supplementary Fig. 34. Temperature-dependent transient PL decay curves under vacuum from 77K~300K (Logarithmic processing of the horizontal coordinate): (a) G1@C[3]A, (b) G2@C[3]A, (c) G3@C[3]A, (d) G4@C[3]A, (e) G5@C[3]A, (f) G6@C[3]A and (g) G7@C[3]A.

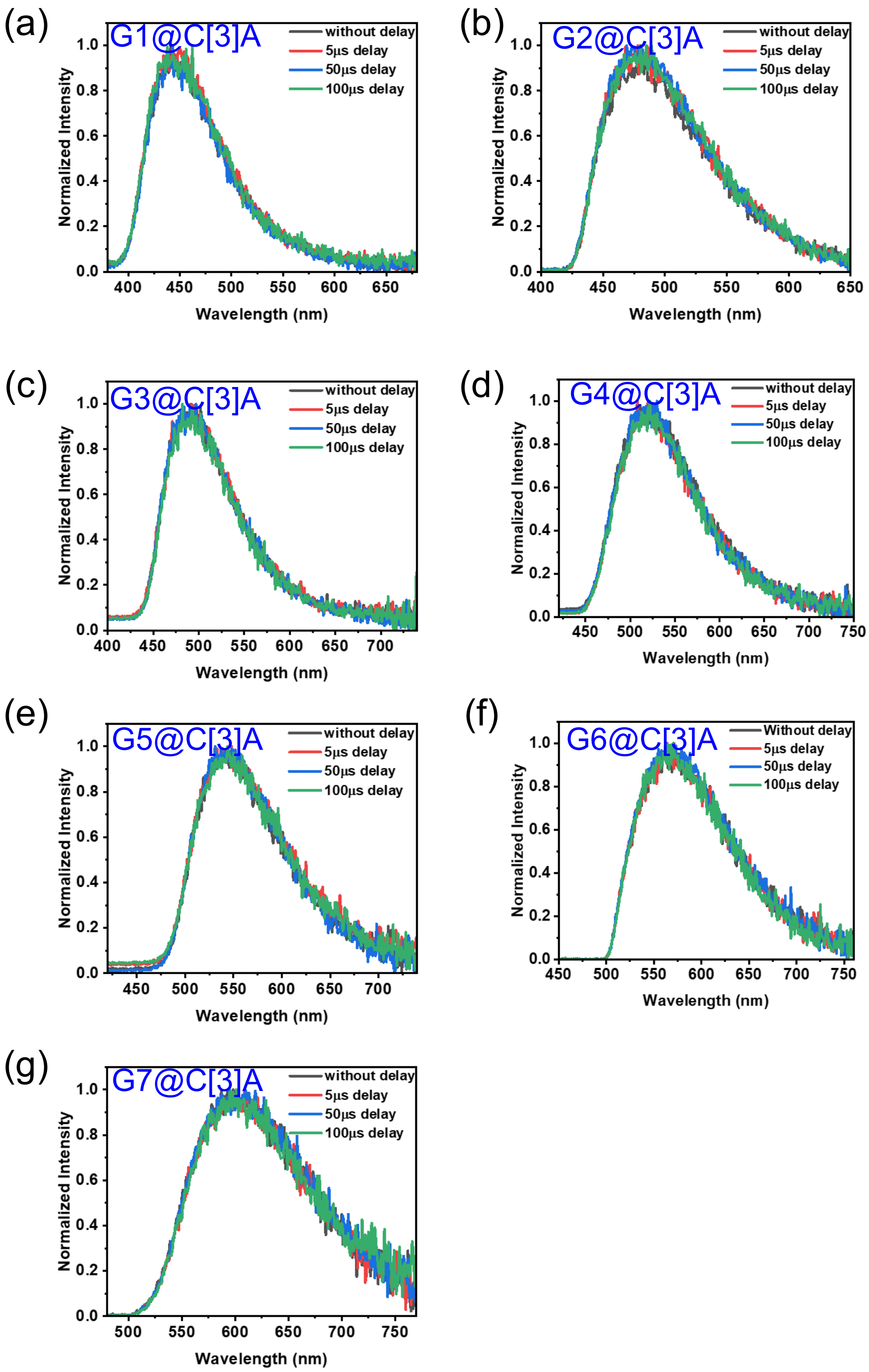

Supplementary Fig. 35. Time-resolved PL spectra under vacuum at 300K: (a) G1@C[3]A, (b) G2@C[3]A, (c) G3@C[3]A, (d) G4@C[3]A, (e) G5@C[3]A, (f) G6@C[3]A and (g) G7@C[3]A.

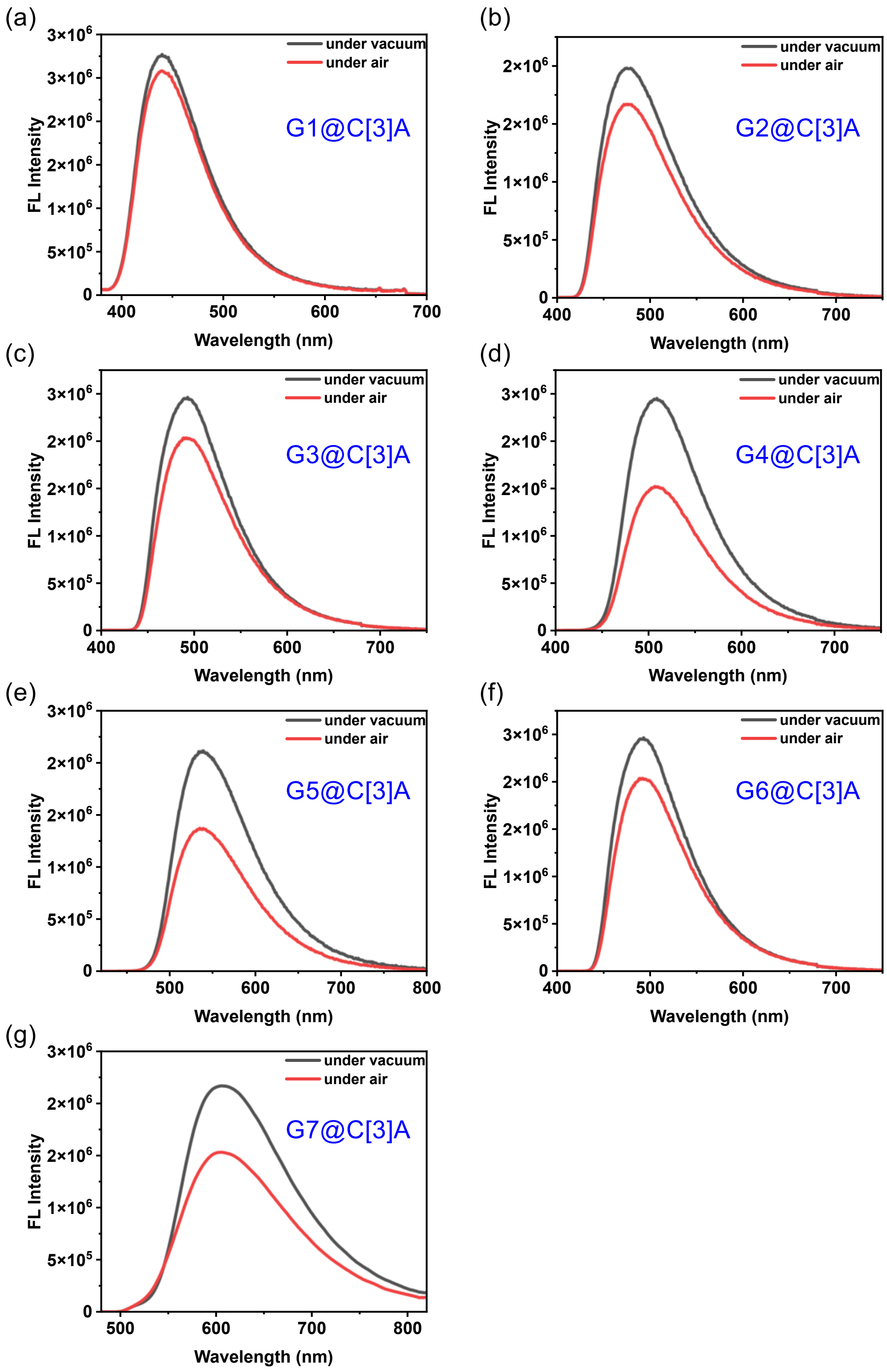

Supplementary Fig. 36. PL spectra in vacuum (dark grey lines) and air (red lines) at 300K: (a) G1@C[3]A, (b) G2@C[3]A, (c) G3@C[3]A, (d) G4@C[3]A, (e) G5@C[3]A, (f) G6@C[3]A and (g) G7@C[3]A.

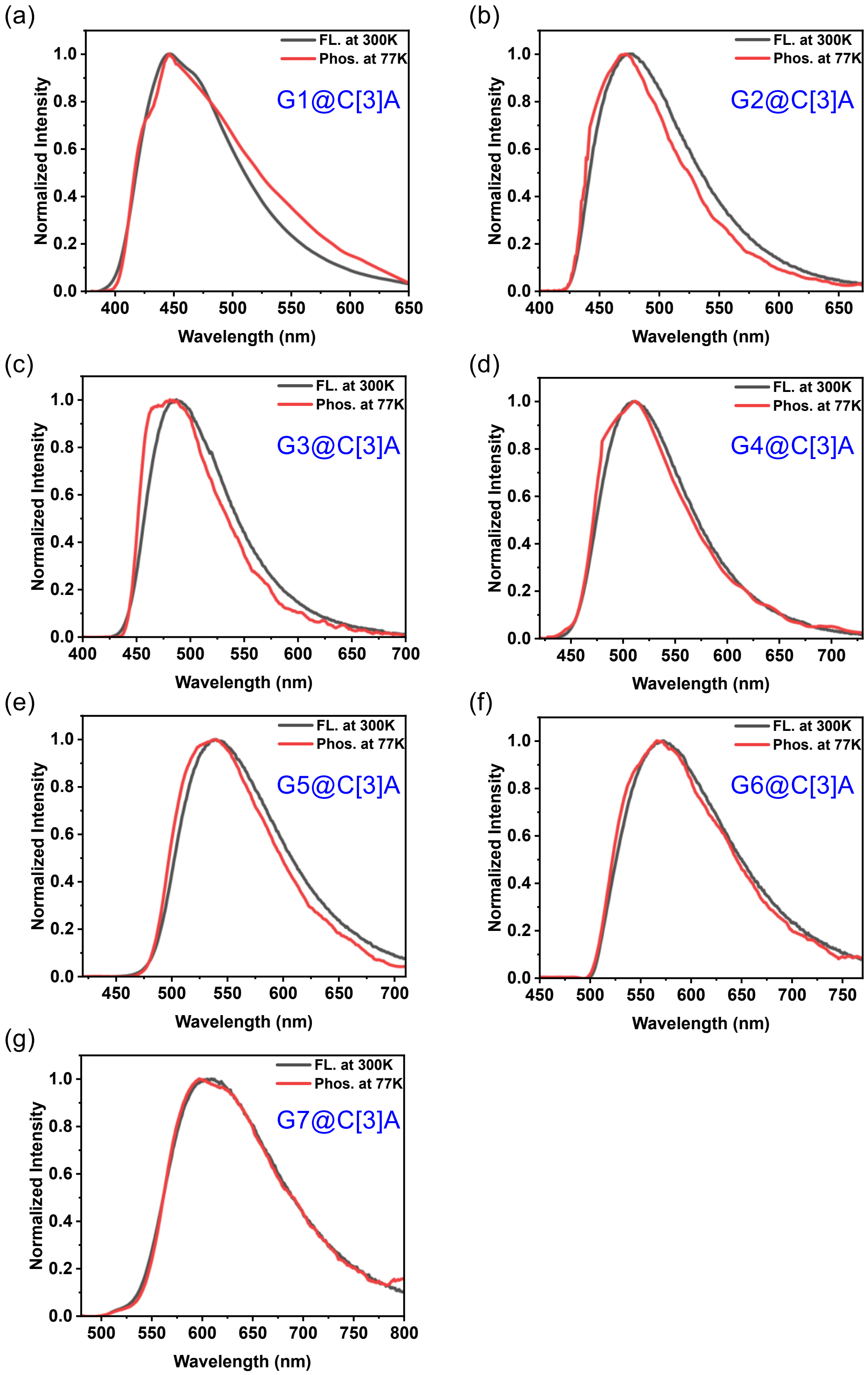

Supplementary Fig. 37. Fluorescence (FL.) spectra at 300 K and phosphorescence (Phos.) spectra under vacuum at 77 K: (a) G1@C[3]A, (b) G2@C[3]A, (c) G3@C[3]A, (d) G4@C[3]A, (e) G5@C[3]A, (f) G6@C[3]A and (g) G7@C[3]A.

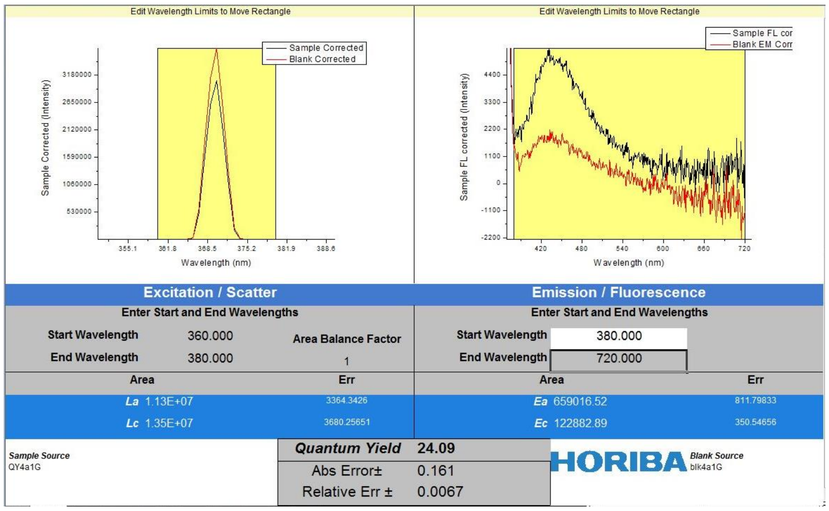

Supplementary Fig. 38. PLQY of G1@C[3]A.

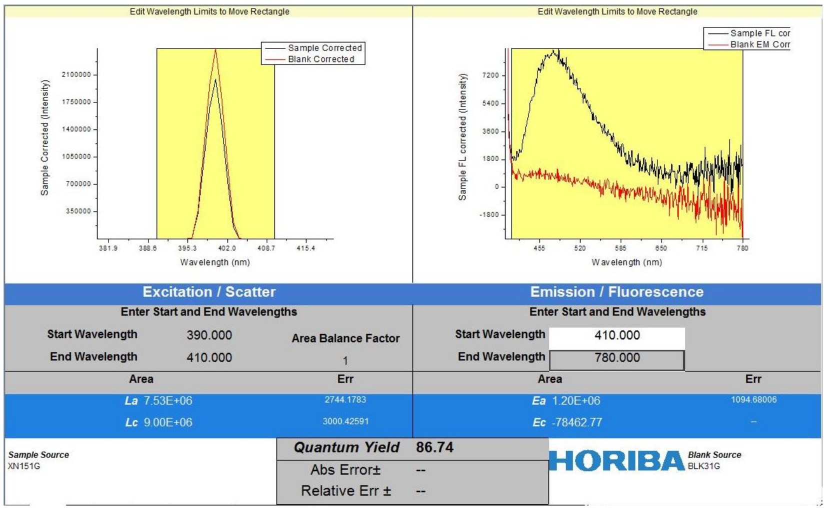

Supplementary Fig. 39. PLQY of G2@C[3]A.

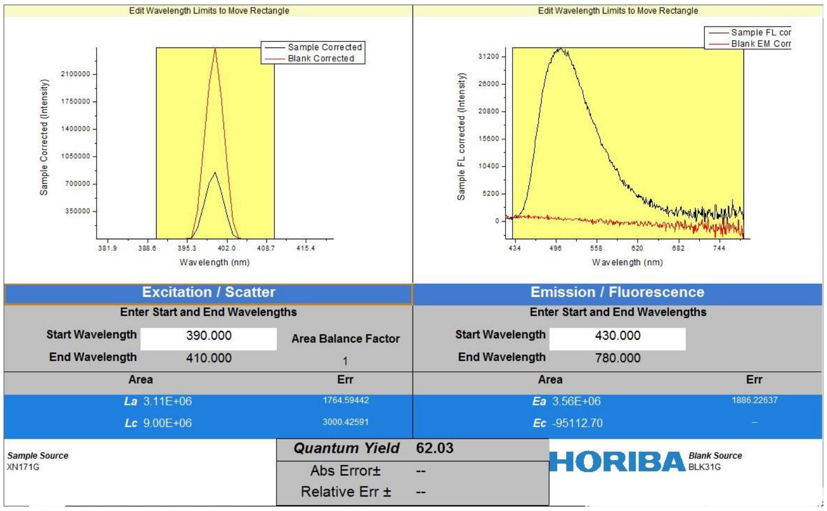

Supplementary Fig. 40. PLQY of G3@C[3]A.

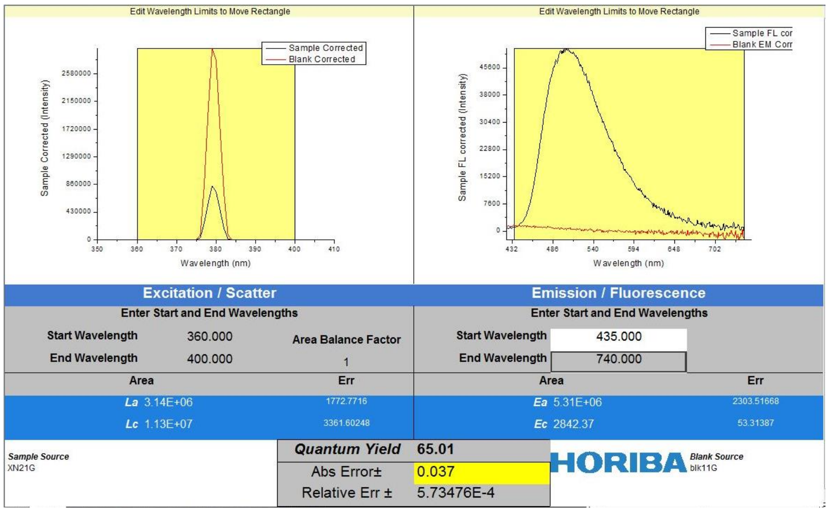

Supplementary Fig. 41. PLQY of G4@C[3]A.

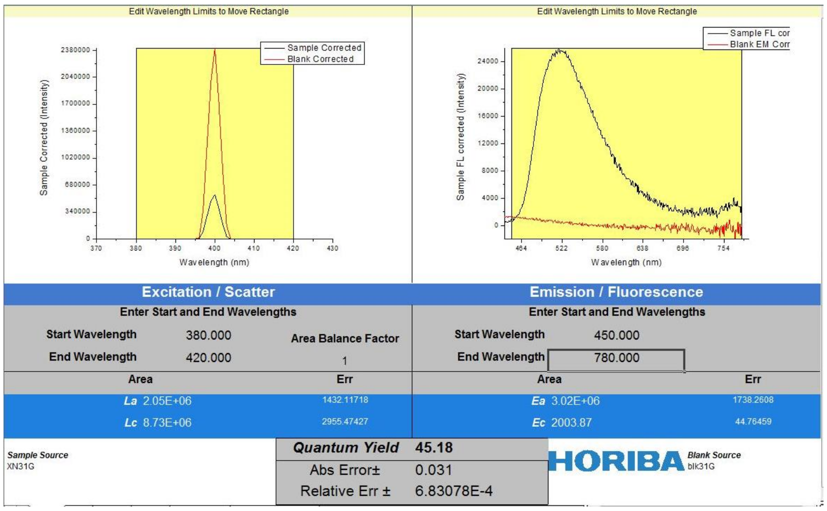

Supplementary Fig. 42. PLQY of G5@C[3]A.

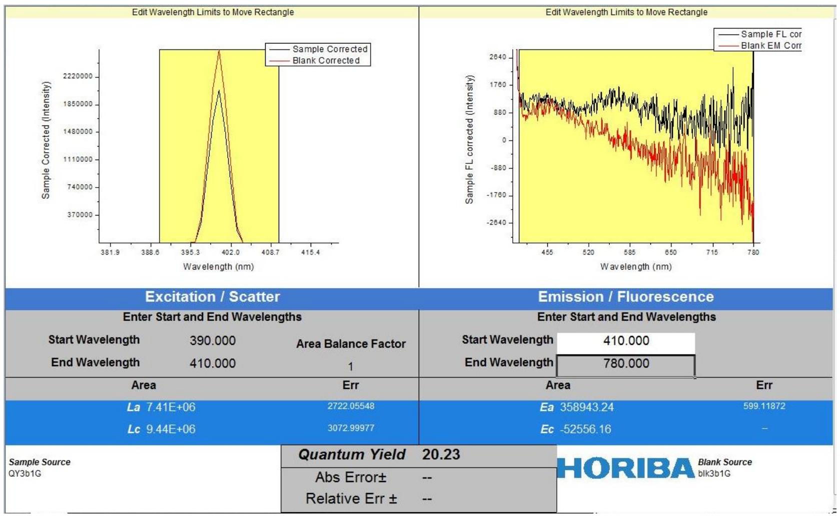

Supplementary Fig. 43. PLQY of G6@C[3]A.

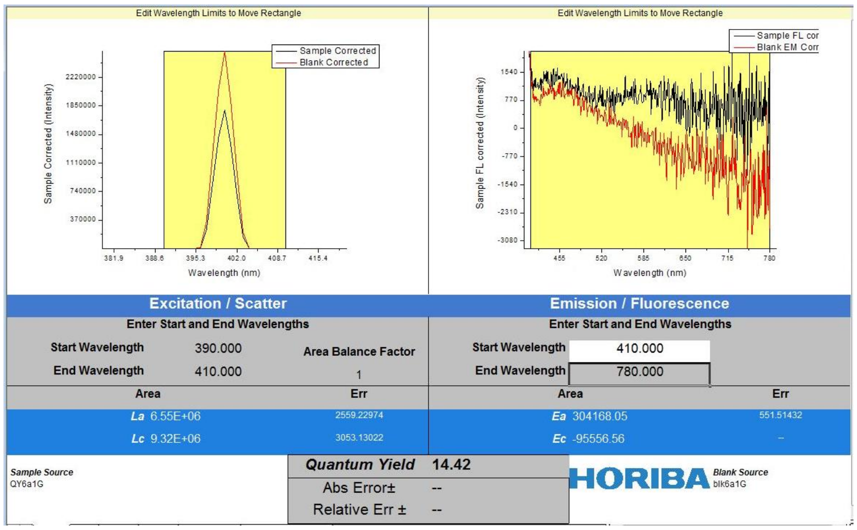

Supplementary Fig. 44. PLQY of G7@C[3]A.

Supplementary Table 9. Summary of photophysical data of G1@C[3]A~G7@C[3]A.

Entry	G1@C[3]A	G2@C[3]A	G3@C[3]A	G4@C[3]A	G5@C[3]A	G6@C[3]A	G7@C[3]A
**CIE coordinate**[a]	(0.17, 0.04)	(0.20, 0.30)	(0.21, 0.40)	(0.29, 0.52)	(0.39, 0.56)	(0.47, 0.52)	(0.58, 0.42)
k_F[b] $[10^5\ s^{-1}]$	22.1	17.1	16.0	1.91	22.8	7.11	2.73
k_{TADF}[c] $[10^5\ s^{-1}]$	0.50	0.72	2.86	2.15	0.17	0.13	0.22
k_{IC}[d] $[10^5\ s^{-1}]$	70.0	2.56	9.81	1.03	72.3	28.4	168
k_{ISC}[e] $[10^6\ s^{-1}]$	1.32	1.53	0.75	1.83	1.36	1.18	3.25
k_{RISC}[f] $[10^4\ s^{-1}]$	1.20	6.24	1.77	14.0	0.40	0.25	0.31
**HOMO**[g] **[eV]**	-4.97	-5.06	-5.09	-5.09	-5.13	-5.14	-5.08
**LUMO**[h] **[eV]**	-1.00	-1.53	-1.78	-1.88	-2.08	-2.05	-2.09
E_g[i] [eV]	3.98	3.54	3.31	3.21	3.05	3.09	2.99
E_S/E_T[j] [eV]	2.963/2.950	2.641/2.639	2.410/2.408	2.365/2.358	2.192/2.191	2.196/2.193	2.059/2.051
ΔE_{ST}[k] [meV]	13	2	2	7	1	3	8

[a]Calculated PL emission color coordinates in the CIE 1931 chromaticity diagram. [b]Rate constant of prompt fluorescence. [c]Rate constant of delayed fluorescence. [d]Rate constant of internal conversion. [e]Rate constant of intersystem conversion. [f]Rate constant of reverse intersystem crossing. [g]HOMO energy levels obtained from theoretical calculation. [h]LUMO energy levels obtained from theoretical calculation. [i]HOMO-LUMO energy gap calculated from energy levels of HOMO and LUMO. [j]S_1 and T_1 energy levels from theoretical calculation. [k]Singlet-triplet energy gap estimated from theoretical calculation. The calculation formulas for the rate constants are expressed as following list:

$k_F = \Phi_F/\tau_F$

$\Phi_{PL} = k_F/(k_F + k_{IC})$

$\Phi_F = k_F/(k_F + k_{IC} + k_{ISC})$

$\Phi_{ISC} = k_{ISC}/(k_F + k_{IC} + k_{ISC})$

$k_{TADF} = \Phi_{TADF}/(k_{ISC} + \tau_{TADF})$

$k_{RISC} = k_F \times k_{TADF} \times \Phi_{TADF}/(k_{ISC} + \Phi_F)$

2.6 Hole-electron Analysis of G1@C[3]A~G7@C[3]A

As a very practical method for revealing the excitation properties of electrons, hole-electron analysis can provide a clear image of hole and electron distribution, which describing the positions and excited electrons leaving and arriving, respectively.

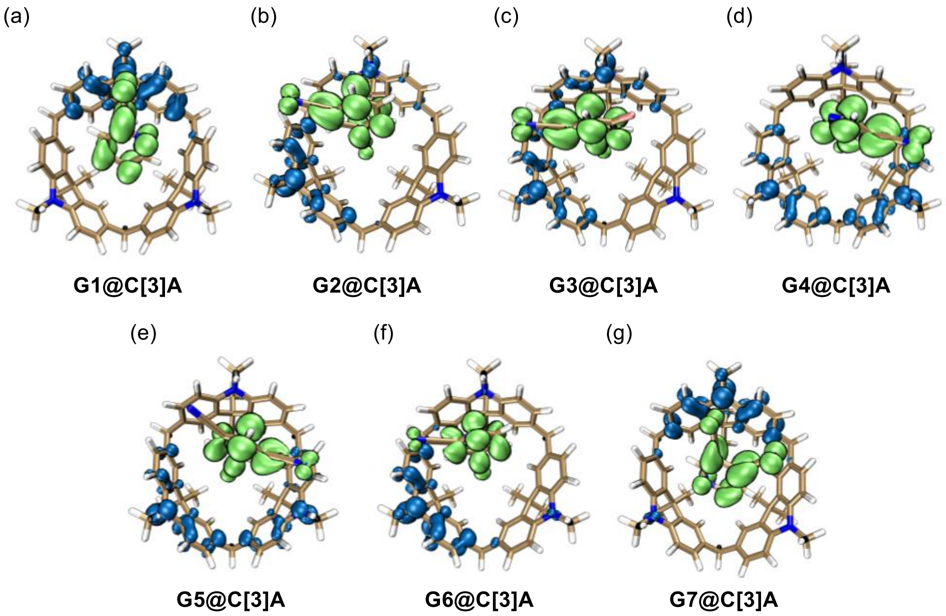

Supplementary Fig. 45. Real space representation of hole and electron distributions of S_0-S_1 excitation (isovalue = 0.002): (a) G1@C[3]A, (b) G2@C[3]A, (c) G3@C[3]A, (d) G4@C[3]A, (e) G5@C[3]A, (f) G6@C[3]A, (g) G7@C[3]A. Blue and green denote the hole and electron distributions.

Supplementary Table 10. Summary of hole-electron analysis characteristic index of excitation (S_0-S_1).

Entry	D_idx (Å)	Sr (a.u.)	t_idx (Å)
**G1@C[3]A**	2.792	0.44874	0.986
**G2@C[3]A**	2.375	0.06560	0.379
**G3@C[3]A**	2.200	0.07295	0.389
**G4@C[3]A**	2.657	0.10285	0.884
**G5@C[3]A**	2.746	0.08936	0.838
**G6@C[3]A**	1.978	0.08233	0.281
**G7@C[3]A**	3.449	0.10099	1.841

D_idx is the hole-electron centroid distance. Sr is the hole-electron overlap integral. t_idx is the CT character. A considerable D_idx, small Sr valve (Max=1) and positive t_idx indicate significant charge transfer characteristic in S_0-S_1 excitation process.

3. Supplementary References

1. Frisch, M. J., Trucks, G. W., Schlegel, H. B., Scuseria, G. E., Robb, M. A. & Cheesman, J. R. Gaussian 09, Revision D.01. Gaussian, Inc., Wallingford CT, (2013).
2. Stephenes, P. J., Devlin, F. J., Chabalowski, C. F. & Frisch, M. J. Ab Initio Calculation of Vibrational Absorption and Circular Dichroism Spectra Using Density Functional Force Fields. *J. Phys. Chem.* **98**, 11623–11627 (1994).
3. Rassolov, V. A., Ratner, M. A., Pople, J. A., Redfern, P. C. & Curtiss, L. A. 6-31G* basis set for third-row atoms. *J. Comput. Chem*. **22**, 976–984 (2001).
4. Versluis, L. & Ziegler, T. The Determination of Molecular Structures by Density Functional Theory. The Evaluation of Analytical Energy Gradients by Numerical Integration. *J. Chem. Phys.* **88**, 322–328 (1988).
5. Perdew, J. P., Burke, K. & Ernzerhof, M. Generalized Gradient Approximation Made Simple. *Phys. Rev. Lett.* **77**, 3865 (1996).
6. Runge, E. & Gross, E. K. U. Density-Functional Theory for Time-Dependent Systems. *Phys. Rev. Lett.* **52**, 997–1000 (1984).
7. Krishnan, R., Binkley, J. S., Seeger, R. & Pople, J. A. Self-consistent molecular orbital methods. XX. A basis set for correlated wave functions. *J. Chem. Phys.* **72**, 650–654 (1980).
8. Lu, T. & Chen, F. Multiwfn: A Multifunctional Wavefunction Analyzer. *J. Comput. Chem.* **33**, 580–592 (2012).
9. Humphrey, W., Dalke, A. & Schulten, K. VMD – Visual Molecular Dynamics. *J. Mol. Graphics*. **14**, 33–38 (1996).
10. Lefebvre, C., Rubez, G., Khartabil, H., Boisson, J.-C., Garacía, J.-C. & Hénon, E. Accurately extracting the signature of intermolecular interactions present in the NCl plot of the reduced density gradient versus electron density. *Phys. Chem. Chem. Phys.* **19**, 17928–17936 (2017).

11. Lu, T. & Chen, Q. Independent gradient model based on Hirshfeld partition: A new method for visual study of interactions in chemical systems. *J. Comput. Chem*. **43**, 539–555 (2022).
12. Liu, Z., Lu, T. & Chen, Q. An sp-hybridized all-carboatomic ring, cyclo[18]carbon: Electronic structure, electronic spectrum, and optical nonlinearity. *Carbon* **165**, 461–467 (2020).
13. Zhou, H.-Y., Zhang, D.-W., Li, M. & Chen, C.-F. A Calix[3]acridan-Based Host-Guest Cocrystal Exhibiting Efficient Thermally Activated Fluorescence. *Angew. Chem., Int. Ed.* **61**, e202117872 (2022).
14. Farrugia, L. J. WinGX and ORTEP for Windows: an update. *J. Appl. Cryst.* **45**, 849–854 (2012).
