# Supplementary material for: A general supramolecular strategy for fabricating full-color-tunable thermally activated delayed fluorescence materials
Source: Nat Commun. 2024 Feb 16;15:1425. doi: 10.1038/s41467-024-45717-x (PMC10873404; doi:10.1038/s41467-024-45717-x)
Supplement: Supplementary file 3 — Description of Additional Supplementary Files [file 41467_2024_45717_MOESM3_ESM.pdf]

Description of Additional Supplementary Files

File Name: Supplementary Data 1
Description: The atomic coordinates of G1@C[3]A obtained by DFT calculation.

File Name: Supplementary Data 2
Description: The atomic coordinates of G2@C[3]A obtained by DFT calculation.

File Name: Supplementary Data 3
Description: The atomic coordinates of G3@C[3]A obtained by DFT calculation.

File Name: Supplementary Data 4
Description: The atomic coordinates of G4@C[3]A obtained by DFT calculation.

File Name: Supplementary Data 5
Description: The atomic coordinates of G5@C[3]A obtained by DFT calculation.

File Name: Supplementary Data 6
Description: The atomic coordinates of G6@C[3]A obtained by DFT calculation.

File Name: Supplementary Data 7
Description: The atomic coordinates of G7@C[3]A obtained by DFT calculation.

File Name: Supplementary Data 8
Description: The atomic coordinates of G1 obtained by DFT calculation.

File Name: Supplementary Data 9
Description: The atomic coordinates of G2 obtained by DFT calculation.

File Name: Supplementary Data 10
Description: The atomic coordinates of G3 obtained by DFT calculation.

File Name: Supplementary Data 11
Description: The atomic coordinates of G4 obtained by DFT calculation.

File Name: Supplementary Data 12
Description: The atomic coordinates of G5 obtained by DFT calculation.

File Name: Supplementary Data 13
Description: The atomic coordinates of G6 obtained by DFT calculation.

File Name: Supplementary Data 14

Description: The atomic coordinates of G7 obtained by DFT calculation.
